# An Insect Herbivore Microbiome with High Plant Biomass-Degrading Capacity

**DOI:** 10.1371/journal.pgen.1001129

**Published:** 2010-09-23

**Authors:** Garret Suen, Jarrod J. Scott, Frank O. Aylward, Sandra M. Adams, Susannah G. Tringe, Adrián A. Pinto-Tomás, Clifton E. Foster, Markus Pauly, Paul J. Weimer, Kerrie W. Barry, Lynne A. Goodwin, Pascal Bouffard, Lewyn Li, Jolene Osterberger, Timothy T. Harkins, Steven C. Slater, Timothy J. Donohue, Cameron R. Currie

**Affiliations:** 1 Department of Energy Great Lakes Bioenergy Research Center, University of Wisconsin-Madison, Madison, Wisconsin, United States of America; 2 Department of Bacteriology, University of Wisconsin-Madison, Madison, Wisconsin, United States of America; 3 Smithsonian Tropical Research Institute, Balboa, Ancon, Panama; 4 Department of Energy Joint Genome Institute, Walnut Creek, California, United States of America; 5 Departamento de Bioquímica, Facultad de Medicina, Universidad de Costa Rica, Ciudad Universitaria Rodrigo Facio, San José, Costa Rica; 6 Centro de Investigaciones en Estructuras Microscópicas, Universidad de Costa Rica, Ciudad Universitaria Rodrigo Facio, San José, Costa Rica; 7 Department of Biochemistry and Molecular Biology, Michigan State University, East Lansing, Michigan, United States of America; 8 Department of Energy Plant Research Laboratory, Michigan State University, East Lansing, Michigan, United States of America; 9 Dairy Forage Research Center, United States Department of Agriculture-Agricultural Research Services (USDA-ARS), Madison, Wisconsin, United States of America; 10 Los Alamos National Laboratory, Biosciences Division, Los Alamos, New Mexico, United States of America; 11 454 Life Sciences, a Roche Company, Branford, Connecticut, United States of America; 12 Roche Diagnostics, Roche Applied Science, Indianapolis, Indiana, United States of America; Stanford University School of Medicine, United States of America

## Abstract

Herbivores can gain indirect access to recalcitrant carbon present in plant cell walls through symbiotic associations with lignocellulolytic microbes. A paradigmatic example is the leaf-cutter ant (Tribe: Attini), which uses fresh leaves to cultivate a fungus for food in specialized gardens. Using a combination of sugar composition analyses, metagenomics, and whole-genome sequencing, we reveal that the fungus garden microbiome of leaf-cutter ants is composed of a diverse community of bacteria with high plant biomass-degrading capacity. Comparison of this microbiome's predicted carbohydrate-degrading enzyme profile with other metagenomes shows closest similarity to the bovine rumen, indicating evolutionary convergence of plant biomass degrading potential between two important herbivorous animals. Genomic and physiological characterization of two dominant bacteria in the fungus garden microbiome provides evidence of their capacity to degrade cellulose. Given the recent interest in cellulosic biofuels, understanding how large-scale and rapid plant biomass degradation occurs in a highly evolved insect herbivore is of particular relevance for bioenergy.

## Author Summary

Leaf-cutter ants form massive subterranean colonies containing millions of workers that harvest hundreds of kilograms of leaves each year. They use these leaves to grow a mutualistic fungus that serves as the colony's primary food source. By farming fungus in specialized garden chambers, these dominant Neotropical herbivores facilitate rapid large-scale plant biomass conversion. Our understanding of this degradation process, and the responsible microbial community, is limited. In this study, we track the degradation of plant polymers in leaf-cutter ant fungus gardens and characterize the microbial community potentially mediating this process. We show that cellulose and hemicelluloses are degraded in the fungus gardens and that a previously unknown microbial community containing a diversity of bacteria is present. Metagenomic analysis of this community's genetic content revealed many genes predicted to encode enzymes capable of degrading plant cell walls. The ability of leaf-cutter ants to maintain an external microbial community with high plant biomass-degrading capacity likely represents a key step in the establishment of these ants as widespread, dominant insect herbivores in the Neotropics. This system is an important model for understanding how microbial communities degrade plant biomass in natural systems and has direct relevancy for bioenergy, given recent interest in cellulosic biofuels.

## Introduction

Plant cell walls contain the largest reservoirs of organic carbon on Earth [1]. This carbon is largely inaccessible to most organisms, occurring in the form of cellulose, hemicelluloses, and lignin. Certain bacteria and fungi are capable of deconstructing these recalcitrant plant polymers, and thus play a critical role in nutrient cycling in the biosphere. Lignocellulolytic microbes form symbiotic relationships with animals that feed on plant biomass, providing their hosts with access to nutrients in return for a constant supply of plant polymers. Recent microbiome studies have revealed how these communities mediate plant biomass deconstruction in animals, including detritivores [2], ruminants [3], and omnivores [4]–[6]. Here, we characterize the microbiome of an important Neotropical herbivore, the leaf-cutter ant *Atta colombica.*

**Figure 1:**
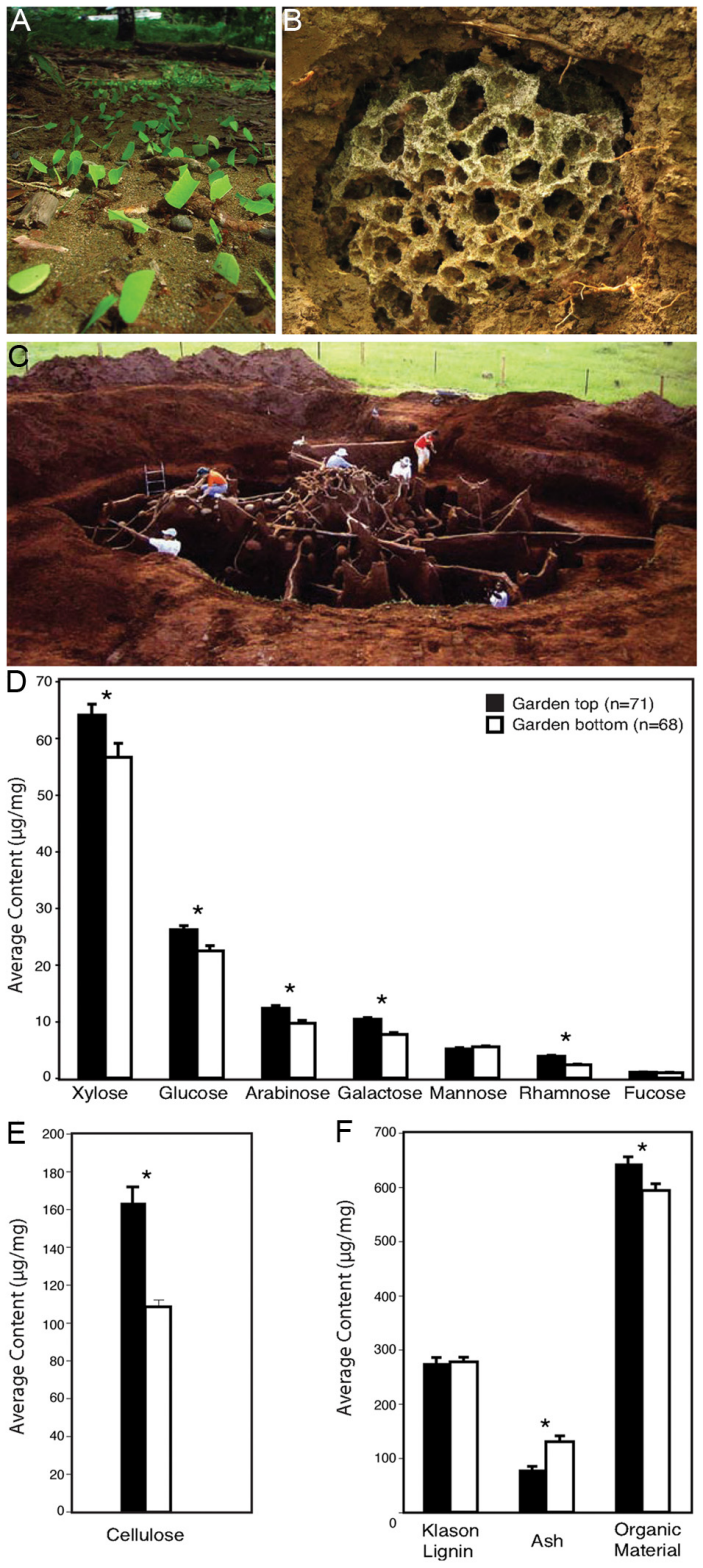
Organic polymer characterization of leaf-cutter ant fungus gardens. Leaf-cutter ants forage for leaves (A) that they use to cultivate a fungus in specialized gardens (B) within their massive colonies (C). Sugar composition analysis of the plant biomass from the top and bottom layers of multiple fungus garden chambers shows an overall decrease in average content for many of the components of hemicellulose (D) and cellulose (E). In contrast, lignin (F) exhibited no change in average content. Error bars in graphs are standard error of the mean. The asterisks indicate a significant decrease in overall average content between top and bottom samples (two-tailed paired *t* test, *P*<0.05). [Photo credits: river of leaves, used under the GNU Free Documentation License CC-BY-SA-3.0,2.5,2.0,1.0; exposed fungus garden, Jarrod J. Scott/University of Wisconsin-Madison; concrete nest, Wolfang Thaler].

Leaf-cutter ants in the genus *Atta* are one of the most conspicuous and widespread insects in the New World tropics, forming massive colonies composed of millions of workers. Mature colonies forage hundreds of kilograms in leaves each year (Figure 1A), substantially altering forest ecosystems and contributing to nutrient cycling [7]. Leaf-cutter ants do not feed directly on harvested leaves; rather, they use leaf fragments as substrate to cultivate a mutualistic fungus in specialized subterranean gardens (Figure 1B and 1C). The fungus serves as the primary food source for the colony and in return is provided with substrate, protection from competitors, and dispersal through colony founding [7]–[9]. Despite the impact of these ants on tropical ecosystems, and the critical role leaves play in *Atta* colonies reaching immense sizes, our current understanding of plant biomass deconstruction within fungus gardens is limited.

## Results/Discussion

The primary function of leaf-cutter ant fungus gardens is to convert plant biomass into nutrients for the ants: it serves as the ants' external digestive system [10]. Fungus gardens have a clear distinction between the top layer, which retains the green, harvested state of plant leaves; and the bottom layer, which contains mature fungus and partially-degraded plant material. This difference is due to the temporal process of plant biomass transformation by the ants; freshly-harvested leaves are integrated into the garden top, while material at the bottom is removed by the ants and placed into specialized refuse dumps. Plant biomass degradation in the garden is thought to be mediated exclusively by the ants' mutualistic fungus (order: Agaricales), but its recently reported inability to degrade cellulose [11] poses the question as to what plant polymers are degraded in the fungus garden matrix. We sampled the top and bottom layers of fungus gardens from five colonies of *Atta colombica* leaf-cutter ants in Gamboa, Panama and performed sugar composition analyses. Our quantification of plant biomass polymer content from these layers revealed that crystalline cellulose and sugars representing various plant polysaccharides, such as hemicelluloses, decreased in content from garden top to bottom (Figure 1D and 1E), whereas lignin did not (Figure 1F). Cellulose in particular, had one of the highest percent decreases, dropping by an average content of 30% from the top to the bottom of the garden.

Our finding that certain plant cell wall polymers are consumed in the fungus garden, including cellulose, which is not known to be degraded by the fungal cultivar, suggests that other microbes may be partially responsible for this deconstruction; a prediction consistent with previous reports of cellulase activity of unknown origin within the fungus garden [12], [13]. We explored this possibility by characterizing the fungus garden microbial communities of three *A. colombica* leaf-cutter ant colonies using near-full length 16S rDNA clone sequencing, short-read SSU rDNA pyrotag sequencing, and whole community metagenome sequencing. A total of 703 and 2,794 near full-length bacterial 16S rDNA sequences were generated for fungus garden top and bottom layers, respectively (Table S1), and short-read pyrotag sequencing of the same samples yielded 8,968 and 11,362 sequences, respectively. PCR using full-length Archaea-specific primers failed to amplify Archaeal 16S rDNA. Community metagenome sequencing of whole fungus gardens using pyrosequencing [14] generated over 401 Mb of sequence (Table S2), and assembly resulted in 155,000 contigs and 200,000 singletons, totaling 130 Mb.

**Figure 2:**
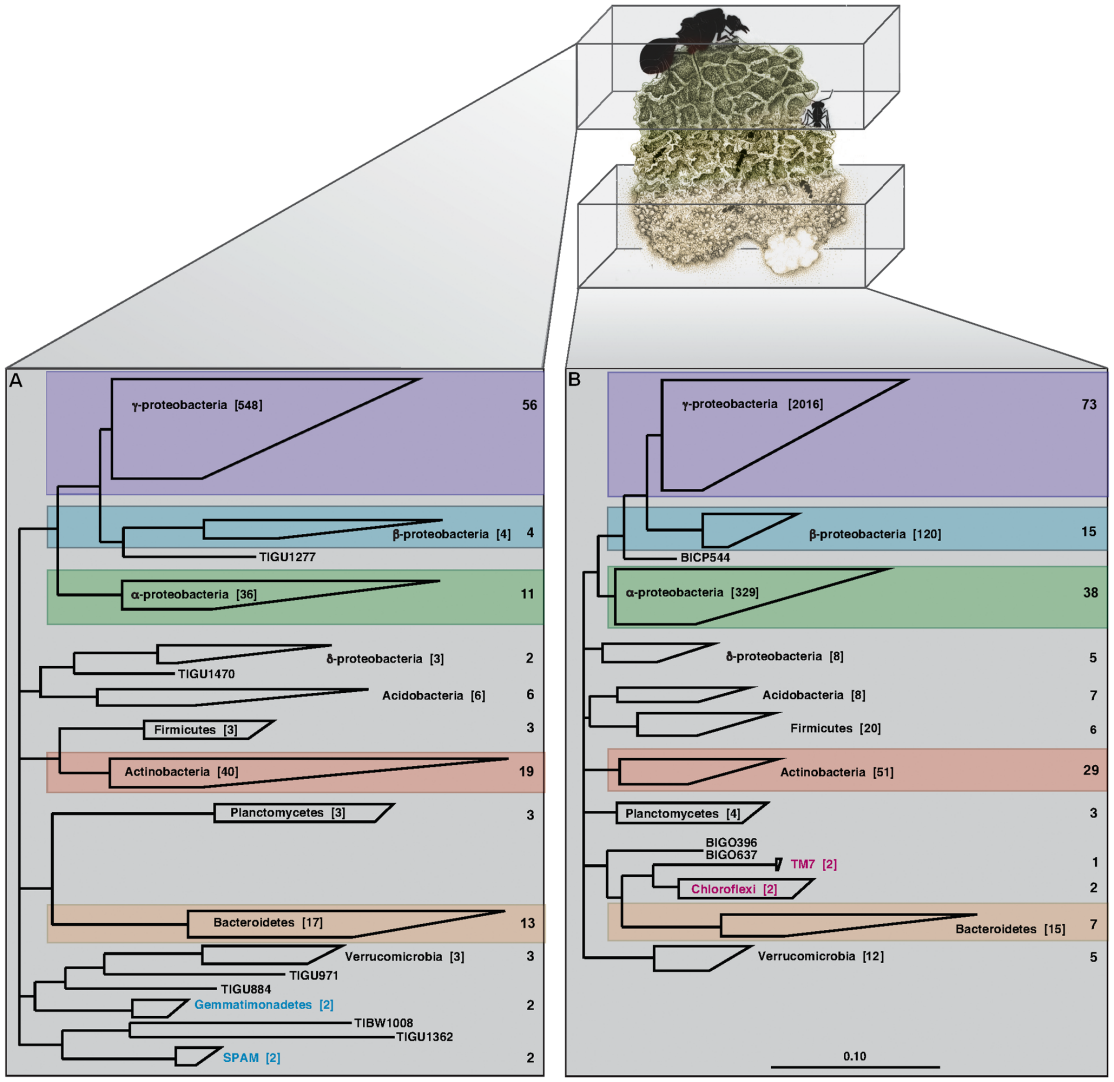
Phylogenetic analysis of the leaf-cutter ant fungus garden. A phylogenetic analysis of near-full length 16S rDNA sequence libraries from the top (A) and bottom (B) layers of leaf-cutter ant fungus gardens was performed. Identified phylotypes were tabulated and mapped to their respective phyla as shown. Total numbers of phylotypes are shown to the right of each phylum, and the total number of clones for each phylum is shown in square brackets. Comparison of top and bottom layers indicates that leaf-cutter ant fungus gardens are dominated by phylotypes belonging to the α-proteobacteria, β-proteobacteria, γ-proteobacteria, Actinobacteria, and the Bacteroidetes as highlighted. Phylotypes belonging to specific phyla were found exclusive to top and bottom samples, including the Gemmatimonadetes and candidate phylum SPAM (blue lettering) in the top, and the Chloroflexi and candidate phylum TM7 (red lettering) in the bottom of the garden.

These DNA sequences indicate the presence of a diverse community of bacteria in leaf-cutter ant fungus gardens (Figure 2, Figure S1, Figure S2). Full-length 16S rDNA libraries contained 132 phylotypes (97% sequence identity) from 9 phyla in garden tops (Figure 2A, Table S3), and 197 phylotypes from 8 phyla in garden bottoms (Figure 2B, Table S3). Comparison of the phylogenetic diversity between top and bottom layer samples using UniFrac [15] indicates that the top layer diversity is different from bottom layer diversity (Figure S3). Both top and bottom layers were dominated by phylotypes in the α-proteobacteria, β-proteobacteria, γ-proteobacteria, Actinobacteria, and Bacteroidetes (Figure 2 and Figures S4, S5, S6, S7, and S8), which collectively contributed 80% (117 of 148 phylotypes) and 85% (185 of 217 phylotypes) of the bacterial diversity detected from top and bottom samples, respectively. A comparison of total generated sequences from these phyla further confirms that these phylotypes are abundant, with 92% (645 of 703 clones) and 91% (2540 of 2794 clones) of all sequenced clones belonging to these 5 lineages for top and bottom samples, respectively. Data from 16S rDNA short-read sequences also confirmed these findings, and further revealed rare phylotypes not found in the full-length analysis, including members of the candidate phyla NC10 [16], OP10 [17], and TM6 [18] (Table S4). Bacterial diversity comparisons among colonies and vertical layers revealed a number of consistent phylotypes, the majority of which are γ-proteobacteria (Figure S9, Figure S10, Table S5). Interestingly, the full-length 16S rDNA libraries revealed phylotypes in the Gemmatimonadetes and candidate phylum SPAM [19] (2 phylotypes each; Figure 2A) exclusive in garden tops, whereas phylotypes in the Chloroflexi and candidate phylum TM7 [20] (1 phylotype each; Figure 2B) were only detected in the garden bottoms. The short-read 16S rDNA sequences confirmed these findings (Table S4), suggesting that specific phyla may play specialized roles within vertical layers of the garden.

Our phylotype diversity analyses were further confirmed through community metagenomics, which does not suffer from the PCR bias inherent to 16S rDNA sequencing [21]. Phylogenetic binning of our community metagenome (Table 1 and Table S6) using a number of different approaches including the program PhymmBL [22], indicates that the fungus garden is dominated by γ-proteobacteria (30% of total bacterial sequences), α-proteobacteria (16%), Actinobacteria (9%), δ-proteobacteria (7%), and β-proteobacteria (7%) (Figure S11, Table S7, Text S1). In particular, the most highly represented sequences are from γ-proteobacterial genera in the family *Enterobacteriaceae*. Our phylogenetic binning analysis also revealed DNA sequences predicted to be derived from insects, fungi, and plants (Figure S12, Table S2, Table S8, Text S1). It is likely that these sequences originate from the ants, their fungal symbiont, and their primary plant feedstuffs, although genome sequences are currently not available for comparison.

**Table 1:** Top 25 ranks and total nucleotide counts of the leaf-cutter ant fungus garden metagenome as phylogenetically binned using the complete microbial genome collection and PhymmBL.

Genus	Taxonomic Group	Metagenome vs. Genome Collection (nucleotide)	Metagenome vs. Genome Collection (protein)	PhymmBL
*Pantoea*	γ-proteobacteria	1 (535,392)	1 (473,904)	1 (619,953)
*Klebsiella*	γ -proteobacteria	2 (286,032)	2 (199,941)	3 (335,333)
*Bradyrhizobium*	α-proteobacteria	3 (109,462)	5 (128,838)	11 (110,268)
*Serratia*	γ -proteobacteria	4 (81,025)	11 (66,510)	53 (22,643)
*Methylobacterium*	α-proteobacteria	5 (71,411)	7 (86,100)	5(229,988)
*Rhodopseudomonas*	α-proteobacteria	6 (70,871)	17 (52,119)	17 (78,968)
*Streptomyces*	Actinobacteria	7 (69,344)	13 (59,562)	21 (67,358)
*Pseudomonas*	γ -proteobacteria	8 (63,984)	8 (87,396)	10 (118,354)
*Burkholderia*	β-proteobacteria	9 (65,098)	12 (66,453)	2 (417,279)
*Enterobacter*	γ -proteobacteria	10 (72,117)	9 (81,630)	33 (37,067)
*Anaeromyxobacter*	δ-proteobacteria	11 (54,832)	16 (55,095)	41 (31,470)
*Solibacter*	Acidobacteria	12 (47,848)	3 (237,534)	-
*Erwinia*	γ -proteobacteria	13 (39,935)	14 (58,392)	20 (67,526)
*Mycobacterium*	Actinobacteria	14 (42,108)	15 (51,360)	9 (125,575)
*Rhizobium*	α-proteobacteria	15 (36,392)	25 (37,599)	6 (191,857)
*Salmonella*	γ -proteobacteria	16 (35,192)	22 (39,477)	14 (85,169)
*Escherichia*	γ -proteobacteria	17 (52,529)	14 (58,392)	4 (250,623)
*Frankia*	Actinobacteria	18 (34,092)	23 (42,075)	59 (21,300)
*Acidobacterium*	Acidobacteria	19 (32,107)	4 (146,133)	95 (11,575)
*Ralstonia*	β-proteobacteria	20 (30,259)	20 (44,067)	7 (133,715)
*Saccharopolyspora*	Actinobacteria	21 (29,082)	58 (16,947)	110 (8,578)
*Roseiflexus*	Chloroflexi	22 (29,067)	10 (77,787)	183 (2,811)
*Sorangium*	δ-proteobacteria	23 (27,235)	18 (52,308)	29 (46,538)
*Gluconobacter*	α-proteobacteria	24 (26,348)	27 (35,670)	31 (26,501)
*Rhodococcus*	Actinobacteria	25 (22,675)	34 (35,640)	12 (105,948)

To identify how the fungus garden microbial community associated with leaf-cutter ants mediates plant polymer degradation, we performed a carbohydrate-active enzyme (CAZy) [23] characterization of the garden community metagenome. This analysis identified 69 gene modules across 28 families of glycosyl hydrolases, carbohydrate esterases, and polysaccharide lyases (Table 2). In total, 58% of the sequences predicted to code for enzymes putatively involved in plant polymer degradation, including cellulose and hemicellulose, were of bacterial origin. These enzymes include β-mannosidases (GH1), α-galactosidases (GH1, GH4, GH57), and cellulases (β-1,4-glucanase; GH8), suggesting that bacteria are important contributors to plant polymer degradation within leaf-cutter ant fungus gardens.

**Table 2:** Carbohydrate-active enzymes in the leaf-cutter ant fungus garden community metagenome.

CAZy Family*	Known CAZy Activities*	Correlated Pfam†	Fungus Garden Metagenome‡	Source Organisms^∥^
CBM50	peptidoglycan-binding lysin module	LysM Domain	1	1 gamma
GH1	β-glucosidase, β-galactosidase, β-mannosidase, and others	Glyco_hydro_1	14	7 plant, 3 gamma, 1 Thermotoga, 1 Chloroflexi, 1 actino, 1 cyano
GH4	maltose-6-phosphate glucosidase, α-glucosidase, α-galactosidase, and others	Glyco_hydro_4	2	1 Chloroflexi, 1 Clostridia
GH7	endoglucanase, cellobiohydrolase, chitosanase	Glyco_hydro_7	1	1 fungal
GH8	chitosanase, cellulase, licheninase, and others	Glyco_hydro_8	3	1 beta, 2 gamma
GH9	endoglucanase, cellobiohydrolase, β-glucosidase	Glyco_hydro_9	3	3 plant
GH16	xyloglucan, keratan-sulfate endo-1,4-β-galactosidase, endo-1,3-β-glucanase, and others	Glyco_hydro_16	5	5 plant
GH17	glucan endo-1,3-β-glucosidase, glucan 1,3-β-glucosidase, licheninase, and others	Glyco_hydro_17	3	3 plant
GH18	chitinase, endo-β-N-acetylglucosaminidase	Glyco_hydro_18	2	1 delta, 1 plant
GH19	chitinase	Glyco_hydro_19	1	1 plant
GH20	β-hexosaminidase, lacto-N-biosidase, β-1,6-N-acetylglucosaminidase, and others	Glyco_hydro_20	1	1 gamma
GH22	lysozyme type C, lysozyme type I, α-lactalbumin	Lys, C-type lysozyme	1	1 insect
GH24	lysozyme	lysozyme	1	1 gamma
GH26	β-mannanase, β-1,3-xylanase	Glyco_hydro_26	2	1 actino, 1 Deinococcus-Thermus
GH30	glucosylceramidase, β-1,6-glucanase, β-xylosidase	Glyco_hydro_30	1	1 actino
GH31	α-glucosidase, α-1,3-glucosidase, sucrase-isomaltase, and others	Glyco_hydro_31	7	1 fungal, 2 plant, 1 Bacteroides, 3 gamma
GH35	β-galactosidase, exo-β-glucosaminidase	Glyco_hydro_35	1	1 plant
GH37	α,α-trehalase	Trehalase	3	2 insect, 1 gamma
GH47	α-mannosidase	Glyco_hydro_47	1	1 fungal
GH57	α-amylase, 4-α-glucanotransferase, α-galactosidase, and others	Glyco_hydro_57	2	2 Dictyoglomi
GH65	α,α-trehalase, maltose phosphorylase, trehalose phosphorylase, and others	Glyco_hydro_65	2	1 alpha, 1 actino
GH89	α-N-acetylglucosaminidase	α -N-acetyl glucosaminidase	2	2 plant
GH102	peptidoglycan lytic transglycosylase	transglycosylase	1	1 gamma
CE4	acetyl xylan esterase, chitin deacetylase, chitooligosaccharide deacetylase, and others	Polysaccharide deacetylase	4	1 actino, 1 cyano, 1 delta, 1 acido
CE8	pectin methylesterase	Pectinesterase	1	1 actino
CE11	UDP-3-0-acyl N-acetylglucosamine deacetylase	UDP-3-O-acyl N-acetylglycosamine deacetylase	1	1 acido
CE14	N-acetyl-1-D-myo-inosityl-2-amino-2-deoxy-α-D-glucopyranoside deacetylase, diacetylchitobiose deacetylase, mycothiol S-conjugate amidase	GlcNAc-PI de-N-acetylase	2	1 acido, 1 Chloroflexi
PL1	pectate lyase, exo-pectate lyase, pectin lyase	Pec_lyase_C	1	1 gamma

*CAZy: carbohydrate-active enzymes, http://www.CAZy.org.

**†:** Pfam, http://pfam.sanger.ac.uk.

**‡:** Number of detected CAZymes (correlated to Pfams) in the leaf-cutter ant fungus garden metagenome.

^∥^As determined by phylogenetic binning (see Methods for details). Organism designations: alpha, α-proteobacteria; beta, β-proteobacteria; gamma, γ-proteobacteria; delta, δ-proteobacteria; acido, Acidobacteria; actino, Actinobacteria; cyano, Cyanobacteria.

**Figure 3:**
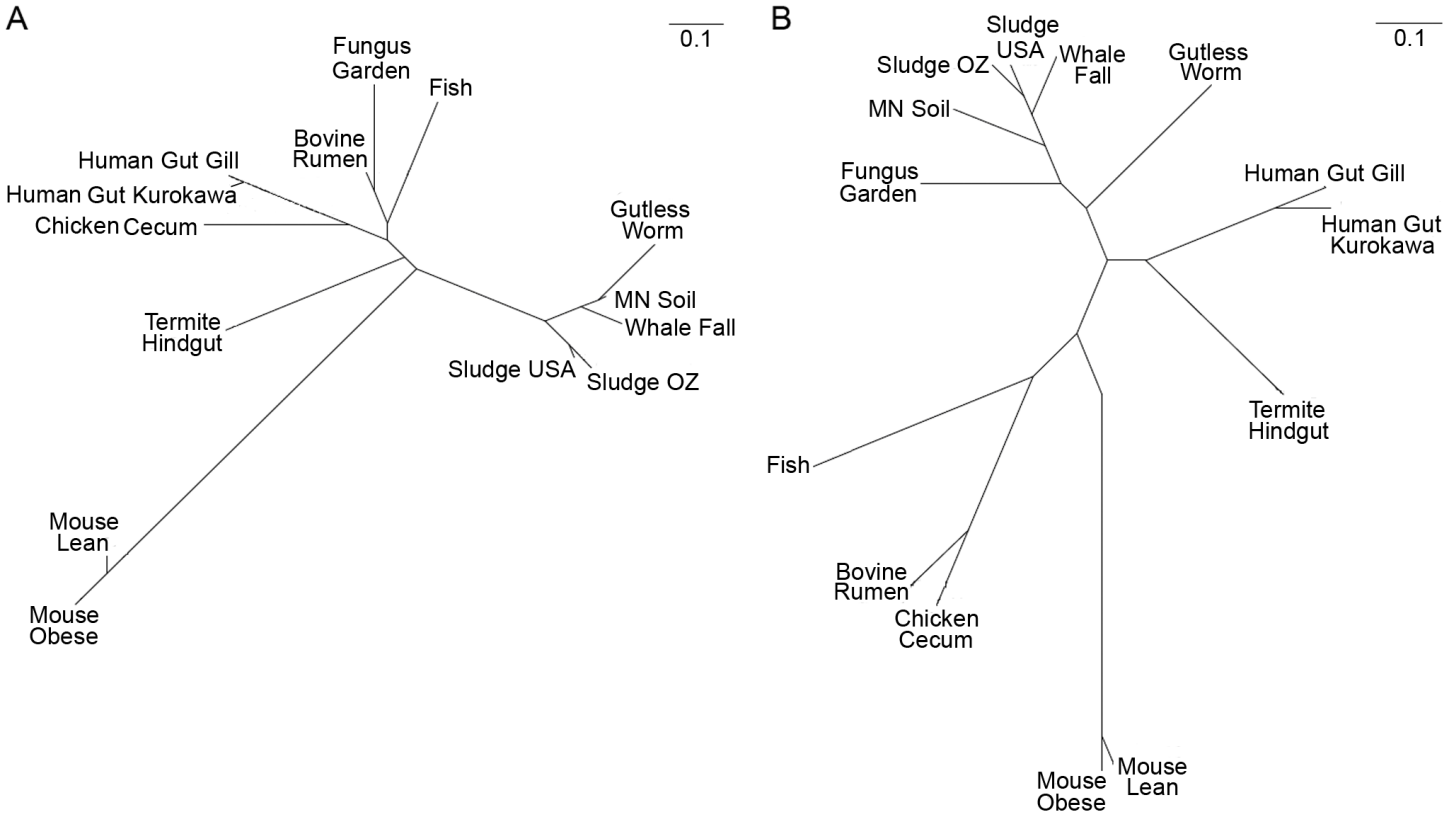
CAZy clustering of the fungus garden metagenome. Comparative clustering of the leaf-cutter ant fungus garden community metagenome with 13 other metagenomes. The predicted proteome from each metagenome was compared using carbohydrate-active enzymes (CAZy) profiles (A) and clusters of orthologous groups (COGs) profiles (B). CAZy and COG profiles for each metagenome was generated and clustered using Pearson's product moment. An unrooted tree (UPGMA) was then generated using PHYLIP and visualized using phylodendron.

We further explored the underlying mechanisms for plant biomass deconstruction in leaf-cutter ants by comparing the predicted bacterial CAZy profile of the fungus garden metagenome with those of 13 other metagenomes from similar environments that exhibit biomass degradation including animal guts and soil. Clustering analysis of these profiles showed that the fungus garden metagenome groups closest to bovine rumen [3] (Figure 3A). Comparison of shared CAZymes between these two metagenomes revealed enzymes involved in amylose (GH57), galactan (GH4), mannan (GH1), maltose (GH65), pectin (CE8), and xylan (CE4, GH26, GH31) deconstruction (Table S9). Many of these oligosaccharide polymers are components of hemicelluloses and other carbohydrates known to be degraded in both bovine rumen [24] and leaf-cutter ant fungus gardens (Figure 1D). Our CAZy profile clustering reveals the importance and similarity of carbohydrate degradation in these two microbiomes, as these metagenomes did not group together in a similar clustering analysis involving entire gene content (Figure 3B, Figure S13, Table S10, Table S11, Text S2).

Despite leaf-cutter ant fungus gardens and bovine rumen utilizing similar plant biomass, leaves and grass, the microbial communities in these systems are markedly different. In the bovine rumen, the majority of resident bacteria are in the genera *Prevotella* (phylum Bacteroidetes), *Fibrobacter* (phylum Fibrobacteres), and *Ruminococcus* (phylum Firmicutes) [25], whereas leaf-cutter ant fungus gardens primarily contain bacteria from the Proteobacteria (Figure 2, Table S7). The similarity in carbohydrate-degrading potential between these two microbiomes is surprising, and the difference in their bacterial communities suggests that there is evolutionary convergence of enzymatic approaches for the deconstruction of at least some plant polymers. Given that there currently are a limited number of plant biomass degrading metagenomes available for comparison, and that the microbiomes used in our analysis were generated using different sequencing technologies and DNA extraction methods, which we are unable to account for (a difficulty that has been previously noted [26]), it is likely that future work may reveal other microbiomes exhibiting CAZyme profiles more similar to leaf-cutter ant fungus gardens than the bovine rumen. Nevertheless, this analysis provides insights into how two microbial communities that utilize similar plant biomass deconstruct polysaccharides.

To further examine the role of cellulolytic bacteria in leaf-cutter ant fungus gardens we characterized representative isolates of *Klebsiella* and *Pantoea*, the two most abundant bacterial genera identified in our community metagenome (Table 1, Table S6). We sequenced the genomes and analyzed the predicted proteomes of *Klebsiella variicola* At-22 and *Pantoea* sp. At-9b (Table S12); two isolates obtained from the fungus gardens of *Atta cephalotes* leaf-cutter ants. Both genomes contained a number of sequences predicted to code for enzymes known to be involved in plant polymer degradation, including cellulases (β-1,4-glucanase; GH8), β-galactosidases (GH2), chitinases (GH18), α-xylosidases (GH31), α-mannosidases (GH47), α-rhamnosidases (GH78), and pectinesterases (CE8) (Table S13, Table S14). Bioassays on pure cultures of these bacteria further revealed their capacity to degrade cellulose (Table S15), suggesting that *Klebsiella* and *Pantoea* may play a role as cellulose-degrading symbionts in the gardens of leaf-cutter ants. The symbiosis between these bacteria and leaf-cutter ants is further supported by previous work, which showed they can be consistently isolated from fungus gardens across the diversity and geography of leaf-cutter ants [10]. Indeed, these bacteria appear to be responsible for a significant amount of the nitrogen that is fixed in leaf-cutter fungus gardens; nitrogen that has been shown to be integrated into the ants [10]. Our finding that *Klebsiella* and *Pantoea* are the most abundant bacteria present in the gardens of *A. colombica*; genomic and physiological support for their capacity to degrade cellulose; and previous reports of their contributions to fixed nitrogen in leaf-cutter ant fungus gardens, provides evidence that these bacteria are important symbionts of leaf-cutter ants.

**Figure 4:**
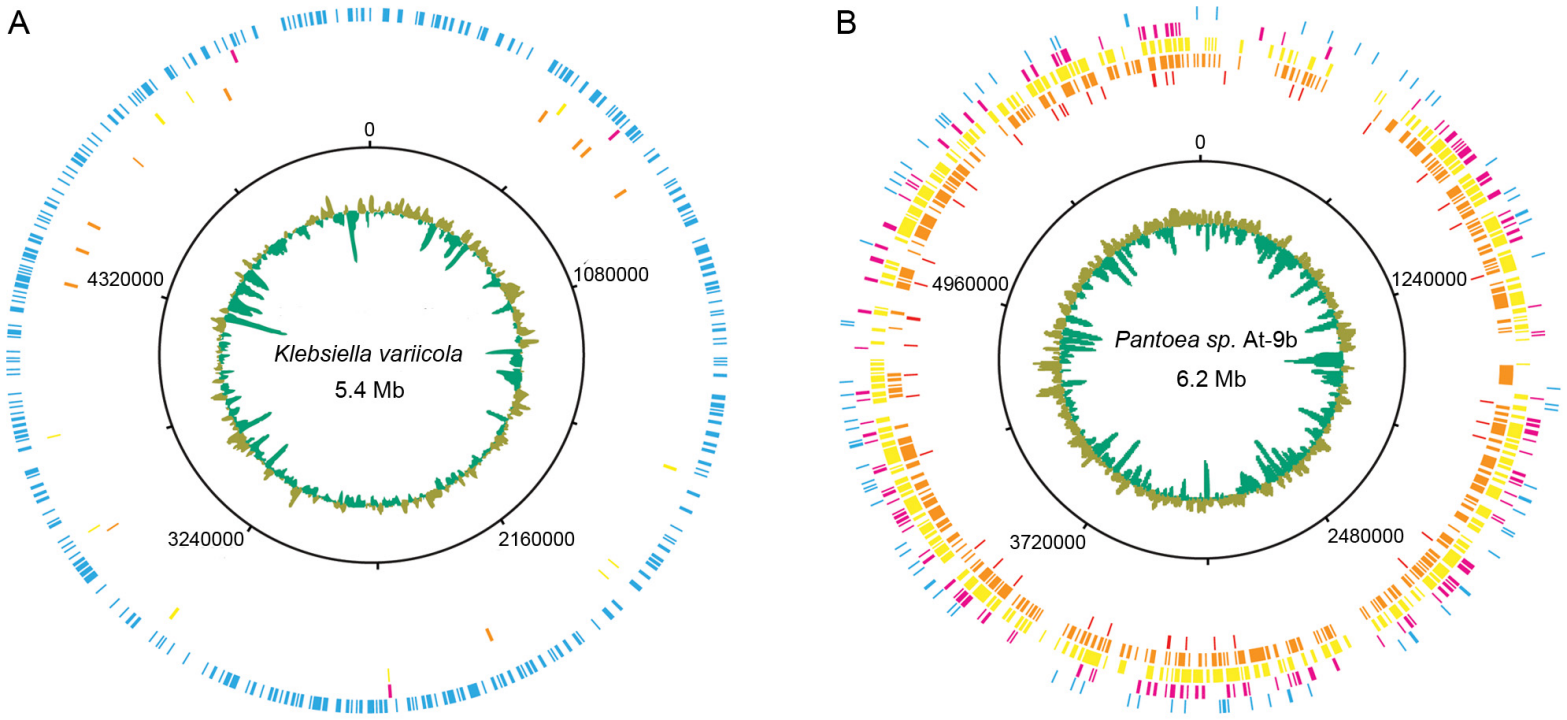
Leaf-cutter ant fungus garden metagenome recruitment analysis. Leaf-cutter ant fungus garden metagenome recruitment analysis. Reads from the leaf-cutter ant fungus garden community metagenome are shown mapped onto the draft genome sequences of the two leaf-cutter ant bacterial symbionts *Klebsiella variicola* At-22 (A) and *Pantoea* sp. At-9b (B). The sequence identity of each recruited read is as follows: blue, 95%–100%; magenta, 90%–95%; yellow, 85%–90%; gold, 80%–85%, and red, 75%–80%. The draft genomes are represented as concatenated contigs in order of decreasing size, and the corresponding coordinates are shown in the second-most inner ring. The average GC content for these draft genomes are shown in the innermost ring with green representing above-average GC content, and olive representing below-average GC content.

Because our fungus garden metagenome and *Klebsiella* and *Pantoea* genomes originate from different *Atta* species, we examined the potential strain diversity of these symbionts by performing a recruitment analysis [27]. This was done by comparing the community metagenome reads against the microbial genome collection and our *Klebsiella* and *Pantoea* genomes (Figure 4A and 4B). Of all 887 genomes analyzed, the genus *Pantoea* had the highest number of recruited reads (2,064), while *Klebsiella* had the third highest (1,226) (Table S16). Mapping of the recruited reads specific to *Klebsiella variicola* At-22 and *Pantoea* sp. At-9b onto their respective genomes showed markedly different results. For *Klebsiella*, 90% of the reads recruited to *Klebsiella variicola* At-22 had sequence identities >98%, indicating that both *Atta* species possess *Klebsiella* symbionts with highly-similar genomes (Figure 4A, Figure S14, Table S16). In contrast, only 4% of the *Pantoea* recruited reads had sequence identities >98% (Figure 4B, Figure S14, Table S16). This supports previous findings that multiple *Pantoea* species exist in leaf-cutter ant fungus gardens [10]. Further comparison of the two γ-proteobacteria GH8 cellulases identified in the community metagenome (Table 2) against the genomes of *Klebsiella variicola* At-22 and *Pantoea* sp. At-9b showed that they matched sequences in these genomes with identities of 99% and 87%, respectively. These data indicate that these two symbionts are present in the fungus gardens of both *Atta* species where they may play a role as cellulose-degrading symbionts.

### Conclusions

Our study presents the first functional metagenomic characterization of the microbiome of an insect herbivore. We reveal that the microbial community within the fungus gardens of leaf-cutter ants contains not only the fungal cultivar, but a diverse assembly of bacteria dominated by γ-proteobacteria in the family *Enterobacteriaceae*. We further show that these bacteria likely participate in the symbiotic degradation of plant biomass in the fungus garden, indicating that the fungal cultivar is not solely responsible for this process, as has been previously assumed. This suggests a model of plant biomass degradation in the fungus garden that includes both bacteria and the fungal cultivar, and we speculate that persistent cellulose-degrading bacterial symbionts like *Klebsiella* and *Pantoea* could work in concert with the fungal cultivar to deconstruct plant polymers.

As an external digestive system, the fungus garden of leaf-cutter ants parallels the role of the gut in other plant biomass degrading systems like bovines and termites. The presence of a bacterial community dominated by Proteobacteria in leaf-cutter ant fungus gardens is similar to the gut microbiota reported for other insect herbivores, suggesting that bacteria in this phylum may be widespread in their association with herbivorous insects [28]–[30]. However, in contrast to other insect herbivores, the external nature of the leaf-cutter ant digestive system removes the restrictions imposed by the physical limitations of internal guts. This feature is likely responsible for these ants achieving massive colony sizes that harvest vast quantities of plant biomass to support their extensive agricultural operations. As a result, these herbivores have a considerable impact on their surrounding ecosystem by contributing significantly to the cycling of carbon and nutrients in the Neotropics. This study of the leaf-cutter ant fungus garden microbiome illustrates how a natural and highly-evolved microbial community deconstructs plant biomass, and may promote the technological goal of converting cellulosic plant biomass into renewable biofuels.

## Materials and Methods

### Sample Collection

A total of 25 fungus gardens from 5 healthy colonies (5 gardens each) of the leaf-cutter ant *Atta colombica* were collected at the end of May and beginning of June, 2008. These colonies are located along Pipeline Road in Soberanía National Park, Panama (latitude 9° 7′ 0″ N, longitude 79° 42′ 0″ W) and designated N9, N11, N12, N13, and N14, respectively. Each fungus garden was vertically cross-sectioned into thirds with the top third designated as the “top” of the garden and the bottom third designated as the “bottom” of the garden. All material was frozen and transported back to the University of Wisconsin-Madison where it was stored at −20°C prior to processing.

### Sugar Composition and Lignin Analysis

From all 5 colonies (3 gardens per colony), 5 independent samples from fungus garden tops and bottoms of each garden were collected for sugar composition analysis. Thus, a total of 75 fungus garden samples each from the top and bottom were used for this part of our study. This material was tested for crystalline cellulose and hemicellulose (matrix polysaccharide) content as follows.

Cellulose content of fungus garden plant biomass was determined by first washing each sample using Updegraff reagent [31], which removes matrix polysaccharides such as hemicelluloses, pectins and amorphous glucan. The remaining residue, containing only crystalline cellulose, was hydrolyzed using Saeman hydrolysis [32]. The resulting glucose monosaccharides were then quantified with an anthrone colourmetric assay as previously described [32].

For the composition of the matrix polysaccharide content, the following components were tested: arabinose, fucose, galactose, glucose, rhamnose, mannose and xylose. Quantification of these sugars were performed by treating finely ground materials with solvents to remove pigments, proteins, lipids, and DNA from the material as previous described [33]. The residue was de-starched with an amylase treatment, resulting in only cell wall material. This material was then treated with 2M trifluoroacetic acid solubilizing the matrix polysaccharides in form of their monosaccharides, and subsequently derivatized into their corresponding alditol-acetates, which were separated and quantified by GC-MS as previously described [34].

The same set of samples used for sugar composition analysis was also used for lignin content analysis, as previously described [35]. Briefly, all samples were dried to 60°C and ground using a 1-mm cyclone mill and analyzed for total non-lignin organic matter, lignin, and ash (organic and inorganic) content. Total carbohydrate content was assessed through a two-step acid hydrolysis with neutral sugars quantified using GC and uronic acids quantified using colorimetry. Klason lignin was quantified from the ash-free residue from the two-step acid hydrolysis. Ash content was quantified by combustion at 450°C for 18 h and the average µg/mg of material was calculated.

### DNA Extraction

Total DNA was extracted in preparation for either 16S rDNA sequencing or community metagenomic sequencing. For 16S rDNA sequencing, a total of 5 gardens each from 3 *Atta colombica* colonies (N9, N11, and N12) were used. A total of 1 g (wet weight) of fungus garden material was sampled from the top layer of each garden corresponding to each colony, for a combined final weight of 5 g of fungus garden material. Total DNA from this sample was then extracted using a MoBio Power Soil DNA Extraction Kit (MOBIO Laboratories, Carlsbad, CA, USA). The same procedures were performed for all fungus garden bottom layer samples for all 3 colonies.

For community metagenomic sequencing, total community DNA was extracted from 5 whole fungus gardens each from all 5 *Atta colombica* colonies used in this study. A total of 1 g of fungus garden material was sampled from top, middle, and bottom layers from all fungus gardens and combined to produce a final sample weight of 75 g. This material was then enriched for bacteria using a modification of a previously-described protocol [36]. Briefly, total fungus garden material was buffered in 1X PBS (137 mM NaCl, 2.7 mM KCl, 10 mM Na_2_HPO_4_, 2 mM KH_2_PO_4_) containing 0.1% Tween and then centrifuged for 5 minutes at 40×g. This resulted in a 3-layer mixture containing leaf-material at the top, fungal mass in the middle, and bacteria at the bottom. The top and middle layers were carefully removed, buffered with 1X PBS containing 0.1% Tween, and washed using the same centrifugation method an additional 3 times. The final mixture was then centrifuged for 30 minutes at 2800×g, re-suspended in 1X PBS containing 0.1% Tween and filtered through a 100 um filter. Total DNA from this resulting sample was then extracted using a Qiagen DNeasy Plant Maxi Kit (Qiagen Sciences, Germantown, MD, USA).

### 16S rDNA Full-Length and Pyrotag Sequencing

Extracted DNA from fungus gardens was PCR amplified (20 cycles) using full-length universal bacterial (27F [5′-AGA GTT TGA TCC TGG CTC AG-3′] and 1391R [5′- GAC GGG CRG TGW GTR CA-3′]) and archaeal (4aF [5′- TCC GGT TGA TCC TGC CRG-3′] and 1391R [5′- GAC GGG CRG TGW GTR CA-3′]) primers and cloned into the pCR4-TOPO vector (Invitrogen) (See http://my.jgi.doe.gov/general/protocols/SOP_16S18S_rRNA_PCR_Library_Creation.pdf). This was then sequenced using standard Sanger-based capillary sequencing and assembled as previously described [2] (http://www.jgi.doe.gov/sequencing/protocols/prots_production.html). These same samples were then pyrosequenced by first PCR amplifying all samples with prokaryote-specific primers corresponding to the V6-V8 region (1492R [5′- TAC GCY TAC CTT GTT ACG ACT T - 3′] and 926F [5′- AAA CTY AAA KGA ATT GAC GG - 3′] fused to 5-base barcodes (reverse primer only) and 454-Titanium adapter sequences) and then sequenced on a Roche 454 FLX GS Titanium pyrosequencer [14]. All 16S rDNA sequences generated in this study are deposited in GenBank with accessions HM545912–HM556124 and HM556125–HM559218 for near full-length 16S rDNA sequences and pyrotagged 16S rDNA sequences, respectively.

### Phylogenetic Analysis of 16S rDNA Sequences

Assembled full-length 16S contigs were first compared against the National Center for Biotechnological Information's (NCBI) non-redundant nucleotide (nt) and environmental nucleotide (env_nt) databases (accessed: 05/01/2009) using BLAST [37] to verify that all sequences were bacterial. A small number of eukaryotic 18S sequences belonging to the fungus the ants cultivate, *Leucoagaricus gongylophorus*, which were likely amplified due to the cross-reactivity of the 16S primers, were removed. No sequences identified as archaeal were detected from our library generated using archaeal-specific primers, and only bacterial sequences were amplified. Sequences were prepared for alignment by orienting each sequence in the same direction using the computer program Orientation Checker [38], putative chimeras were removed using Bellerophon [39], and each set was de-replicated to remove exact duplicates.

Finalized sets for each sample were then analyzed using the ARB [40] software environment as follows. All full-length 16S rDNA sequences were imported and then aligned using the ARB fast-aligner tool [40] against a user-constructed PT-Server (constructed from the SILVA [41] 16S SSU rDNA preconfigured ARB reference database with 7,682 columns and 134,095 bacterial sequences; accessed: 01/15/2009). The full alignment was manually curated using the ARBprimary editor (ARB_EDIT4) in preparation for phylogenetic and community analysis. Once an acceptable alignment was obtained we created a PHYLIP [42] distance matrix in ARB using the filter-by-base-frequency method (column filter; minimal similarity  = 50%; gaps ignored if occurred in >50% of the samples; 1,320 valid columns). The PHYLIP distance matrix was exported to the MOTHUR software package v.1.5.0 [43] for community analysis and OTU designation. Briefly, the distance matrix was read into MOTHUR and clustered using the furthest neighbor algorithm. From here, we performed rarefaction, rank-abundance, species abundance, and shared analyses. Representative sequences from each OTU at 97% were re-imported into ARB for phylogenetic analysis (Figure S4, S5, S6, S7, and S8). We used a Maximum Likelihood (RAxML [44]) method for all phylogenetic analyses (normal hill-climbing search algorithm) and the above-mentioned method for positional filtering. Closest taxonomic assignment of clones was performed using the Ribosomal Database Project (RDP) [45] by comparing sequences against the type strain database (Table S5).

For pyrotagged short-read 16S rDNA sequences, all sequences were compared against the National Center for Biotechnological Information's (NCBI) non-redundant nucleotide (nt) and environmental nucleotide (env_nt) databases (accessed: 05/01/2009) using BLASTN. Sequences were then classified as either bacterial, archaeal, or eukaryotic, and only those bacterial sequences (20,330) were retained for further analysis.

These sequences were then processed through Orientation Checker, chimeras removed using the program Mallard [38], and subsequently analyzed using MOTHUR in the following fashion. First the entire dataset was de-replicated to eliminate duplicate sequences. The remaining sequences were aligned in MOTHUR against the Greengenes [46] reference alignment (core_set_aligned.imputed.fasta; 7,682 columns, accessed: 09/11/2009) using the Needleman alignment method with the following parameters: k-tuple size  = 9; match  = +1; mismatch penalty  = −3; gap extension penalty  = −1; gap opening penalty  = −5. Sequences were then screened to eliminate those shorter than 400 bp (gaps included). Filtration eliminated 7,062 columns resulting in a total alignment size of 620 bp (gaps included). The remaining dataset was again de-replicated to eliminate duplicate sequences and we constructed a furthest-neighbor distance matrix in MOTHUR using the twice de-replicated, filtered, alignment. All subsequent analyses (rarefaction, rank-abundance, species abundance, and shared analyses) were performed in MOTHUR using this distance matrix.

### UniFrac Analysis

A UniFrac [15] analysis was performed on all full-length 16S rDNA samples generated in this study, including 3 from the top and 3 from the bottom of fungus gardens. MOTHUR was used to generate phylip distance matrices and the computer program Clearcut [47] was then employed to construct neighbor-joining trees. UniFrac was then used to compare these samples as shown in Figure S3.

### Community Metagenome Sequencing and Assembly

Whole community DNA was used to create a shotgun library which was then sequenced using a single pyrosequencing plate on a Roche 454 FLX GS Titanium sequencer. Assembly of the data was performed using the 454 de novo assembler software with default parameters. Total amounts of data generated and statistical coverage is presented in Table S2. Raw sequence reads generated for this microbiome are deposited in NCBI's Short Read Archive under Study Accession SRP001011.1, and assembled contigs and singletons have been deposited into DDBJ/EMBL/GenBank under the accession ADWX00000000.

### Community Metagenome Phylogenetic Binning

The complete set of assembled contigs and singletons representing the fungus garden community metagenome was phylogenetically binned using the following approach. First, the metagenome was compared against NCBI's non-redundant nucleotide (nt) and environmental nucleotide (env_nt) databases (accessed: 05/01/2009) using BLASTN (e-value: 1e-05) and the top hit was retained. The designated phylogenetic classification of the top hit for each contig and singleton was then assessed and binned into one of the following 4 sets: Bacterial, Eukaryotic, Viral, or Unknown. We then performed in-depth phylogenetic binning of the bacterial portion of the fungus garden community metagenome using the current microbial genome collection (http://www.ncbi.nlm.nih.gov/genomes/lproks.cgi, accessed: 05/15/2009). We reasoned that using the current microbial genome collection is a likely a more accurate metric for classifying the bacterial set at the genus level because each genome in this collection is correctly annotated and the current iteration of this collection contains both phylogenetic breadth and depth for many represented genera. As a result, we performed two different phylogenetic bins using the current microbial genome collection.

First, GeneMark [48] was used to predict open reading frames and their corresponding translated proteins of the bacterial portion of the fungus garden community metagenome using a generic bacterial gene model. This predicted proteome was then compared against a local database containing all proteomes in the current microbial genome collection (http://www.ncbi.nlm.nih.gov/genomes/lproks.cgi, accessed: 05/15/2009) supplemented with the predicted proteomes of two bacterial strains (*Klebsiella variicola* At-22 and *Pantoea* sp. At-9b, see below) isolated from the fungus gardens of a related leaf-cutting ant species, *Atta cephalotes*. Comparison of the fungus garden proteome against our microbial reference database was done using BLASTP (e-value: 1e-05) and the phylogenetic identity of the top hit was recorded. The total number of proteins was then tabulated to the genus level. Total nucleotide coding content for each predicted protein was then calculated to determine the total amount of nucleotide represented in each bin.

Second, we performed phylogenetic binning on the bacterial portion of the fungus garden metagenome using the entire nucleotide content of the current microbial genome collection (http://www.ncbi.nlm.nih.gov/genomes/lproks.cgi, accessed: 05/15/2009), and again supplemented with the nucleotide content from the draft genome sequences of our two bacterial isolates from *Atta cephalotes* leaf-cutter ant fungus gardens. Using complete nucleotide content of the current microbial genome collection is advantageous because it includes both coding and intergenic regions, and provides a more robust measure of phylogenetic identity. We compared the entire bacterial portion of the fungus garden metagenome against this database using BLASTN (e-value: 1e-05) and the phylogenetic identity of the top hit was recorded. The total number of contigs and singletons was then tabulated to the genus level and the corresponding nucleotide amounts were also calculated. Furthermore, we performed this same analysis using all high-quality reads from our fungus garden community metagenome. Finally, we employed the phylogenetic binning program PhymmBL [22], which resulted in similar phylogenetic binning results as our comparison against the sequenced genome collection.

### GC Content Analysis

We performed GC content analysis on the Bacterial, Eukaryotic, and Unclassified phylogenetic bins of the leaf-cutter ant fungus garden community metagenome. For the bacterial set, we divided the sequences according to the NCBI Taxonomic Groups Acidobacteria, Actinobacteria, α-proteobacteria, Bacteroidetes, β-proteobacteria, and γ-proteobacteria. We then calculated their GC content, and tabulated the total number of sequences within each group corresponding to each percentage as shown in Figure S11. For Eukaryotic sequences, these were divided into fungal, metazoan, and plant classifications and GC content analysis was also performed as shown in Figure S12. Furthermore, this same analysis was performed for the unclassified portion of the community metagenome and plotted alongside our Eukaryotic GC content analysis.

### Carbohydrate-Active Enzyme Annotation Analysis

The predicted proteome from the bacterial portion of the fungus garden community metagenome was annotated using the carbohydrate active enzyme (CAZy) database [23] as follows. A local database of all proteins corresponding to each CAZy family from the CAZy online database (http://www.cazy.org/, accessed: 06/01/2009) was constructed, and this was used to align the predicted proteome of the bacterial portion of the fungus garden community metagenome using BLASTP (e-value of 1e-05). This proteome was then annotated against the protein family (Pfam [49]) database (ftp://ftp.ncbi.nih.gov/pub/mmdb/cdd/, accessed: 05/01/2009) using RPSBLAST [50] (e-value: 1e-05). A CAZy to Pfam correlation list was then compiled based on the secondary annotations provided through the CAZy online database. Finally, only those proteins that had significant BLAST hits to a protein from our local CAZy database and its corresponding Pfam were retained and designated as a carbohydrate-associated enzyme.

A similar process was performed using the eukaryotic portion of the fungus garden metagenome. However, because of the difficulty in accurately predicting proteins from this subset, due to the lack of good gene models, we compared the contigs and singletons in this subset to our local CAZy and Pfam databases using BLASTX (e-value: 1e-05). Only those hits with significant matches to a protein from our local CAZy database, and its corresponding Pfam were retained and designated as a carbohydrate-associated enzyme in this set.

### Comparative COG and CAZy Cluster Analysis

To determine the similarity of the fungus garden community metagenome with respect to other sequenced metagenomes, we performed a comparative analysis using protein domain and carbohydrate enzyme content as a comparative metric, as previously described [51]. In general, the predicted proteome from the bacterial portion of the fungus garden metagenome was annotated according to clusters of orthologous groups (COGs [52]) database (ftp://ftp.ncbi.nih.gov/pub/mmdb/cdd/, accessed: 05/01/2009) using RPSBLAST (e-value: 1e-05). The predicted proteomes from the following 13 metagenomes were also annotated in the same manner: bovine rumen [3], chicken cecum [53], fish gut and slime [54], gutless worm [55], human gut (Gill) [6], human gut (Kurokawa) [56], Minnesota soil [51], lean mouse [5], obese mouse [5], termite hindgut [2], wastewater sludge USA [57], sastewater sludge OZ [57], and whale fall [51]. The COG profiles from all of these metagenomes were divided according to their COG gene category designations and plotted as a proportion as shown in Figure S13. Cluster analysis of COG profiles for these metagenomes were performed as follows. A matrix was generated with each row corresponding to a metagenome and each column corresponding to a COG ID. The proportion of each COG with respect to the total number of annotated COGs in that metagenome was calculated and populated in the appropriate cell of the matrix. Spearman's rank correlation was then applied to this matrix to generate a similarity matrix correlating each metagenome to each other based on the similarity of each metagenome's COG profile. A distance matrix was then calculated using the neighbor program from the computer suite Phylip [42] (using the UPGMA method), and the resulting unrooted tree was visualized using the phylodendron tree drawing program (http://iubio.bio.indiana.edu/treeapp/, accessed 07/25/2009). This same analysis was also performed using protein domains (Pfam) and no discernable difference in metagenome groupings was detected (data not shown).

A similar approach was used for clustering these metagenomes according to CAZy content. Each metagenome's predicted proteome was annotated using CAZy and correlated to its Pfam annotation as described above. Because each protein potentially encodes for domain that belong to multiple CAZy families (i.e. a protein may contain both a GH and a CBM), we assigned multiple CAZy annotations to a particular protein. A carbohydrate enzyme matrix was then constructed with each row corresponding to a metagenome sample and each column corresponding to a CAZy family. Each cell in this matrix was then populated with the proportion of each CAZy family with respect to the total number of annotated CAZy families in each respective metagenome. Generation of an unrooted tree using this matrix was then constructed using the same procedure outlined for clustering based on the protein domain content metric.

### Draft Genome Sequencing, Assembly, and Annotation

Pure isolates of *Klebsiella variicola* At-22 and *Pantoea* sp. At-9b were cultured from the fungus gardens of the leaf-cutter ant *Atta cephalotes*, as previously described [10]. Genomic DNA from these isolates were extracted, as previously described [10]. Draft genomes of *Klebsiella variicola* At-22 and *Pantoea sp*. At-9b were sequenced at the U.S. Department of Energy Joint Genome Institute (JGI) using a random shotgun approach through a combination of 454 standard and paired-end pyrosequencing (454 Life Sciences, a Roche Company) and 36 bp read Illumina sequencing (Illumina, Inc.). Sequencing using 454 was performed to an average depth of coverage of 30X for both *Klebsiella* and *Pantoea*. All general aspects of library construction and sequencing performed at the JGI can be found at http://www.jgi.doe.gov. A draft assembly for *Klebsiella variicola* At-22 was compiled based on 459,192 reads; for *Pantoea sp.* At9b, a draft assembly was constructed using 557,748 reads. The Phred/Phrap/Consed software package (http://www.phrap.com) was used for sequence assembly and quality assessment of both drafts [58]–[60]. After the shotgun stage, reads were assembled with parallel Phrap (High Performance Software LLC). Automated annotation of these draft genomes were performed by the Computational Biology and Bioinformatics Group of the Biosciences Division of the U.S. Department of Energy Oak Ridge National Laboratory as described at http://genome.ornl.gov/. The draft genome sequence and annotation for *Klebsiella variicola* At-22 and *Pantoea* sp. At-9b were deposited in GenBank under accession numbers CP001891 and ACYJ00000000, respectively.

### Recruitment Analysis

The full set of reads used for the assembly of the fungus garden community metagenome was used to generate a recruitment plot against the draft genomes of *Klebsiella variicola* At-22 and *Pantoea* sp. At-9b, two isolates we cultured from the fungus garden of the leaf-cutter ant *Atta cephalotes* [10], as previously described [27] . Briefly, the contigs from each draft genome were concatenated together in ascending size to produce a “pseudogenome”, and the reads from the fungus garden community metagenome were aligned against a database containing both pseudogenomes, and all genomes from the current microbial genome collection (http://www.ncbi.nlm.nih.gov/genomes/lproks.cgi, accessed: 05/15/2009) using BLASTN. The top hit for each read was retained, and categorized to each genome. We then mapped reads corresponding to *Klebsiella variicola* At-22 and *Pantoea* sp. At-9b onto each organism's respective psuedogenome and further binned them according to their sequence identities as follows: 95%–100%, 90%–95%, 85%–90%, 80%–85%, and 70%–80%. Visualization of the mapped reads onto each respective draft genome was performed using the DNAPlotter software package [61].

### CAZy Analysis of Draft Genomes

A CAZy analysis was performed on the predicted proteomes of *Klebsiella variicola* At-22 and *Pantoea* sp. At-9b using the same approach as described for CAZy analysis of the leaf-cutter ant fungus garden community metagenome. Furthermore, both GH8 cellulases from each of these genomes were compared against the CAZyme of the fungus garden community metagenome at the nucleotide level using BLASTN (e-value: 1e-05).

### Cellulose Degradation Bioassays

Bioassays were performed on pure cultures of *Klebsiella variicola* At-22 and *Pantoea* sp. At-9b to determine their capacity to degrade cellulose. These include carboxymethyl cellulose (CMC) assays and growth on microcrystalline cellulose. CMC assays were performed as previously described [62]. Briefly, pure cultures of both bacteria were inoculated onto yeast malt extract agar (YMEA, 4 g yeast extract, 10 g Bacto Peptone, 4 g Dextrose, 15 g agar) and grown at 25°C for 2 days. Single colonies were then spotted onto carboxymethyl cellulose plates (15 g agar, 5 g carboxymethyl cellulose [Calbiochem, La Jolla, CA]). Detection of cellulose degradation on CMC was performed using congo red, and the ability of each isolate's capacity for cellulose degradation was measured based on the zone of clearing present on the plate. Growth on microcrystalline cellulose was performed by inoculating 10 µl of pure culture into 150 µl of microcrystalline cellulose broth (1 L water and 5 g cellulose powder microcrystalline cellulose [MP Biomedicals, Solon, OH]) and growth was measured using a DTX 880 Multimode Detector Plate Reader (Beckman Coulter Inc., Fullerton, CA) at an absorbance of 595 for 2 days. Positive growth on microcrystalline cellulose was correlated to an increase in the measured absorbance over this period.

## Supporting Information

Figure S1 Rarefaction analysis of the leaf-cutter ant fungus garden full-length 16S rDNA sequences. The combined samples (a), top layer samples (b), and bottom layer (c) samples are plotted as shown. Observed Operational Taxonomic Unit (OTUs) cutoffs at 0.00 (100%), 0.01 (99%), 0.02 (98%), 0.03 (97%), 0.05 (95%), 0.10 (90%), and 0.20 (80%) are plotted as a function of the number of clones.
(1.72 MB TIF)

Figure S2 Rarefaction analysis of the leaf-cutter ant fungus garden short-read pyrotagged 16S rDNA sequences. The combined samples (a), top layer samples (b), and bottom layer samples (c) are plotted as shown. Observed Operational Taxonomic Unit (OTUs) cutoffs were determined at 0.01 (99%), 0.02 (98%), 0.03 (97%), 0.05 (95%), and 0.10 (90%) are plotted as a function of the number of clones.
(1.14 MB TIF)

Figure S3 Comparison of the microbial communities from leaf-cutter ant fungus garden top and bottom sample. The plot was generated using unweighted UniFrac. GT  =  garden top; GB  =  garden bottom.
(0.37 MB TIF)

Figure S4 Phylogenetic diversity of α-proteobacteria in the leaf-cutter ant fungus garden near-full length 16S rDNA sequence library. The shown phylogram was constructed using Maximum Likelihood analysis (RAxML) with 11 near-full length 16S rDNA sequences from the garden top (green), 36 sequences from the garden bottom (red), and other closest-matching 16S rDNA sequences from the Greengenes database. GenBank Accession numbers are also provided for Greengene sequences.
(2.51 MB TIF)

Figure S5 Phylogenetic diversity of β-proteobacteria in the leaf-cutter ant fungus garden near-full length 16S rDNA sequence library. The shown phylogram was constructed using Maximum Likelihood analysis (RAxML) with 4 near-full length 16S rDNA sequences from the garden top (green), 120 sequences from the garden bottom (red), and other closest-matching 16S rDNA sequences from the Greengenes database. GenBank Accession numbers are also provided for Greengenes sequences
(1.98 MB TIF)

Figure S6 Phylogenetic diversity of γ-proteobacteria in the leaf-cutter ant fungus garden near-full length 16S rDNA sequence library. The shown phylogram was constructed using Maximum Likelihood analysis (RAxML) with 70 near-full length 16S rDNA sequences from the garden top (green), 82 sequences from the garden bottom (red), γ-proteobacterial sequences from previous studies of other leaf-cutter ant fungus gardens (blue), and other closest-matching 16S rDNA sequences from the Greengenes database. GenBank Accession numbers are also provided for Greengene sequences.
(6.30 MB TIF)

Figure S7 Phylogenetic diversity of Actinobacteria in the leaf-cutter ant fungus garden near-full length 16S rDNA sequence library. The shown phylogram was constructed using Maximum Likelihood analysis (RAxML) with 40 near-full length 16S rDNA sequences from the garden top (green), 51 sequences from the garden bottom (red), and other closest-matching 16S rDNA sequences from the Greengenes database. GenBank Accession numbers are also provided for Greengene sequences.
(3.65 MB TIF)

Figure S8 Phylogenetic diversity of Bacteroidetes in the leaf-cutter ant fungus garden near-full length 16S rDNA sequence library. The shown phylogram was constructed using Maximum Likelihood analysis (RAxML) with 17 near-full length 16S rDNA sequences from the garden top (green), 14 sequences from the garden bottom (red), and other closest-matching 16S rDNA sequences from the Greengenes database. GenBank Accession numbers are also provided for Greengene sequences.
(2.17 MB TIF)

Figure S9 Venn diagram representation of full-length 16S rDNA phylotypes across 3 different colonies of the leaf-cutter ant *Atta colombica*. Phylotype clusters at different sequence identities are shown at 100% (a), 99% (b), 98% (c), 97% (d), 95% (e), and 90% (f).
(2.24 MB TIF)

Figure S10 Venn diagram representation of short-read pyrotagged 16S rDNA phylotypes across 3 different colonies of the leaf-cutter ant *Atta colombica*. Phylotype clusters at different sequence identities are shown at 100% (a), 99% (b), 98% (c), 97% (d), 95% (e), and 90% (f).
(2.65 MB TIF)

Figure S11 GC content analysis of the bacterial portion of the leaf-cutter ant fungus garden community metagenome. The % GC of each contig and singleton classified as bacterial was tabulated and graphed according to its taxonomic group. The γ-proteobacteria had the highest number of contigs and reads with a % GC commiserate with sequenced γ-proteobacterial genomes. The Actinobacteria had the highest average % GC, as expected based on the average % GC of sequenced Actinobacterial genomes.
(0.99 MB TIF)

Figure S12 GC content analysis of the eukaryotic and unclassified portion of the leaf-cutter ant fungus garden community metagenome. The % GC of each contig and singleton classified as eukaryotic was tabulated and graphed according to the categories fungi, metazoa, and plants. Calculation of the % GC for the unclassified portion of the leaf-cutter ant fungus garden community metagenome is also shown.
(0.70 MB TIF)

Figure S13 Clusters of orthologous groups (COG) analysis of the leaf-cutter ant fungus garden community metagenome compared to 13 other metagenomes. Shown is the number of COG-annotated proteins in each category, represented as a proportion of each metagenome's total COG-annotated proteins for 12 categories. Abbreviations for each metagenome are as follows: chicken cecum (CHC), cow rumen (CRU), fish (FSH), leaf-cutter ant fungus garden (LFG), gutless worm (GWO), human gut - Gill study (HGG), human gut - Kurokawa study (HGK), Minnesota soil (MNS), mouse lean (MLE), mouse obese (MOB), sludge Australia (SOZ), sludge USA (SUS), termite hindgut (THG), and whale fall (WHF).
(1.05 MB TIF)

Figure S14 Average sequence identity of leaf-cutter ant fungus garden community metagenome reads mapped onto complete genomes in the microbial genome collection and the draft genomes of the leaf-cutter ant-associated *Klebsiella variicola* At-22 and *Pantoea* sp. At-9b. Only those organisms with more than 100 mapped reads are shown. The total number of mapped reads is also listed in parentheses beside each organism's name. Average sequence identities are highlighted for *Klebsiella variicola* At-22 (yellow) and *Pantoea* sp. At-9b (orange). Standard deviation bars are also shown.
(2.06 MB TIF)

Table S1 Summary statistics for near full-length and pyrotag 16S rDNA sequencing of leaf-cutter ant fungus gardens. Sequences were generated for garden top and bottom samples from 3 *Atta colombica* leaf-cutter ant colonies. Average sequence length and the total number of sequences generated are also shown.
(0.03 MB DOC)

Table S2 Summary statistics for the leaf-cutter ant fungus garden community metagenome. Raw sequence reads were generated using 454 titanium pyrosequencing and assembled into contigs using only high-quality reads. Reads that could not be assembled were assigned as singletons. Phylogenetic binning of all contigs and singletons were performed using BLAST and comparing against NCBI's non-redundant nucleotide (nt) database to classify into one of bacterial, eukaryotic, viral, unclassified sets.
(0.03 MB DOC)

Table S3 Total phylotypes counts for the leaf-cutter ant fungus garden near full-length 16S rDNA library. Phylotypes are at the genus level (97% identity), and classified at the family and taxonomic groups for top, bottom, and combined samples.
(0.13 MB DOC)

Table S4 Total phylotype counts for the leaf-cutter ant fungus garden short-read pyrotagged 16S rDNA library. Phylotypes were determined by sequence comparison against the Greengenes database (97% sequence identity), and tabulated according to NCBI's Taxonomic Group designation. Total phylotypes across all taxonomic groups are displayed for garden top, bottom and the total combined samples.
(0.05 MB DOC)

Table S5 Phylotypes shared across the top and bottom fungus garden layers of three leaf-cutter colonies (N9, N11, and N12). Phylotypes were clustered at a sequence identity of 97% and four comparisons are shown: N11-N12-N9, N11-N12, N11-N9, and N12-N9. A representative clone from each phylotype cluster was used to determine its classification using the type strain collection in the Ribosomal Database Project (RDP). The length of each representative clone, its RDP classification (Genbank identifier in parenthesis) and its RDP sequence identity score are also shown.
(0.10 MB DOC)

Table S6 Comparison of the top 25 phylogenetic ranks as determined using either the contigs/singletons or reads from the leaf-cutter ant fungus garden metagenome. For reference, binning of the metagenome (contigs/singletons) against the complete microbial genome collection is shown. The rank of each phylogenetic bin and its corresponding nucleotide count is shown.
(0.07 MB DOC)

Table S7 Represented microbial taxonomic groups in the leaf-cutter ant fungus garden community metagenome. The bacterial portion of the fungus garden metagenome was compared against NCBI's non-redundant nucleotide (nr) database and the total amount of sequence corresponding to each taxonomic group was retained and shown. The percentage of each taxonomic group's represented sequence in the total bacterial portion of the fungus garden community metagenome is also shown. A second phylogenetic binning using the computer program PhymmBL was also performed and produced similar results as shown.
(0.05 MB DOC)

Table S8 Top 20 eukaryotic phylogenetic bins of the leaf-cutter ant fungus garden metagenome as determined by comparison against NCBI's non-redundant nucleotide database (nt). Ranks are determined by the highest total nucleotide coverage at the genus level (Shown in parenthesis after each taxa). The classification designation for each genus is also shown.
(0.04 MB DOC)

Table S9 Comparison of the leaf-cutter ant fungus garden metagenome against those of 13 other metagenome using carbohydrate-active enzyme (CAZy) profiles. Shown is the total proportion of CAZy-annotated enzymes (confirmed by Pfam), by family, in each metagenome's predicted CAZyme. Abbreviations are as follows: chicken cecum (CHC), cow rumen (CRU), fish (FSH), leaf-cutter ant fungus garden (LFG), gutless worm (GWO), human gut - Gill study (HGG), human gut - Kurokawa study (HGK), Minnesota soil (MNS), mouse lean (MLE), mouse obese (MOB), sludge Australia (SOZ), sludge USA (SUS), termite hindgut (THG), and whale fall (WHF).
(0.21 MB DOC)

Table S10 Gene category distribution of the bacterial portion of the leaf-cutter ant fungus garden metagenome as annotated using clusters of orthologous groups (COGs). A total of 8,092 ORFs (or ∼50%) out of 16,342 predicted bacterial ORFs in the fungus garden community metagenome was annotated to a COG category, as shown. The % of annotated ORFs for each COG category is also shown.
(0.06 MB DOC)

Table S11 Identified COGs in the leaf-cutter ant fungus garden metagenome that belong to secondary metabolites biosynthesis, transport and catabolism (Q) category. The COG ID, total identified number, and COG annotation are shown.
(0.08 MB DOC)

Table S12 Draft genome characteristics of the leaf-cutter ant-associated nitrogen-fixing bacterial symbionts *Pantoea* sp. At-9b and *Klebsiella variicola* At-22.
(0.03 MB DOC)

Table S13 Carbohydrate-active enzyme (CAZy) annotation of the predicted proteome of *Klebsiella variicola* At-22. Only those proteins that had a significant hit (e-value < 1e-05) to an enzyme in the CAZy database and to each CAZy family's associated protein domain (Pfam) annotation were retained. Specifically, the locus, predicted CAZy family, and top BLAST hit (including closest matching organism) are provided below.
(0.07 MB DOC)

Table S14 Carbohydrate-active enzyme (CAZy) annotation of the predicted proteome of *Pantoea* sp. At-9b. Only those proteins that had a significant hit (e-value < 1e-05) to an enzyme in the CAZy database and to each CAZy family's associated protein domain (Pfam) annotation were retained. Specifically, the locus, predicted CAZy family, and top BLAST hit (including closest matching organism) are provided below.
(0.06 MB DOC)

Table S15 Cellulose-degradation bioassays for *Klebsiella variicola* At-22 and *Pantoea* sp. At-9b. Cultures of both bacteria were grown on carboxymethyl cellulose or microcrystalline. Confirmation of this assay was done by growing these cultures using only crystalline cellulose (CMC) or microcrystalline cellulose as a carbon source. CMC data is reported as the area zone of clearing when assayed using Congo Red (mm2). Microcrystalline cellulose growth is reported as either a plus (+) or minus (−) indicating positive or negative results for growth.
(0.03 MB DOC)

Table S16 Recruitment analysis of the leaf-cutter ant fungus garden community metagenome. Reads from the fungus garden community metagenome were recruited onto complete genomes in the prokaryotic genome collection in addition to the draft genomes of *Klebsiella variicola* At-22 and *Pantoea* sp. At-9b generated in this study. Only those organisms with more than 100 recruited reads are shown. The total number of recruited reads, the number of reads with >98% sequence identity, and the corresponding percentage is shown.
(0.05 MB DOC)

Text S1 GC Content Analysis of the Community Metagenome.
(0.03 MB DOC)

Text S2 COG Clustering Analysis of the Community Metagenome.
(0.04 MB DOC)
