# Supplementary figures and images for: An Insect Herbivore Microbiome with High Plant Biomass-Degrading Capacity
Source: PLoS Genet. 2010 Sep 23;6(9):e1001129. doi: 10.1371/journal.pgen.1001129 (PMC2944797; doi:10.1371/journal.pgen.1001129)

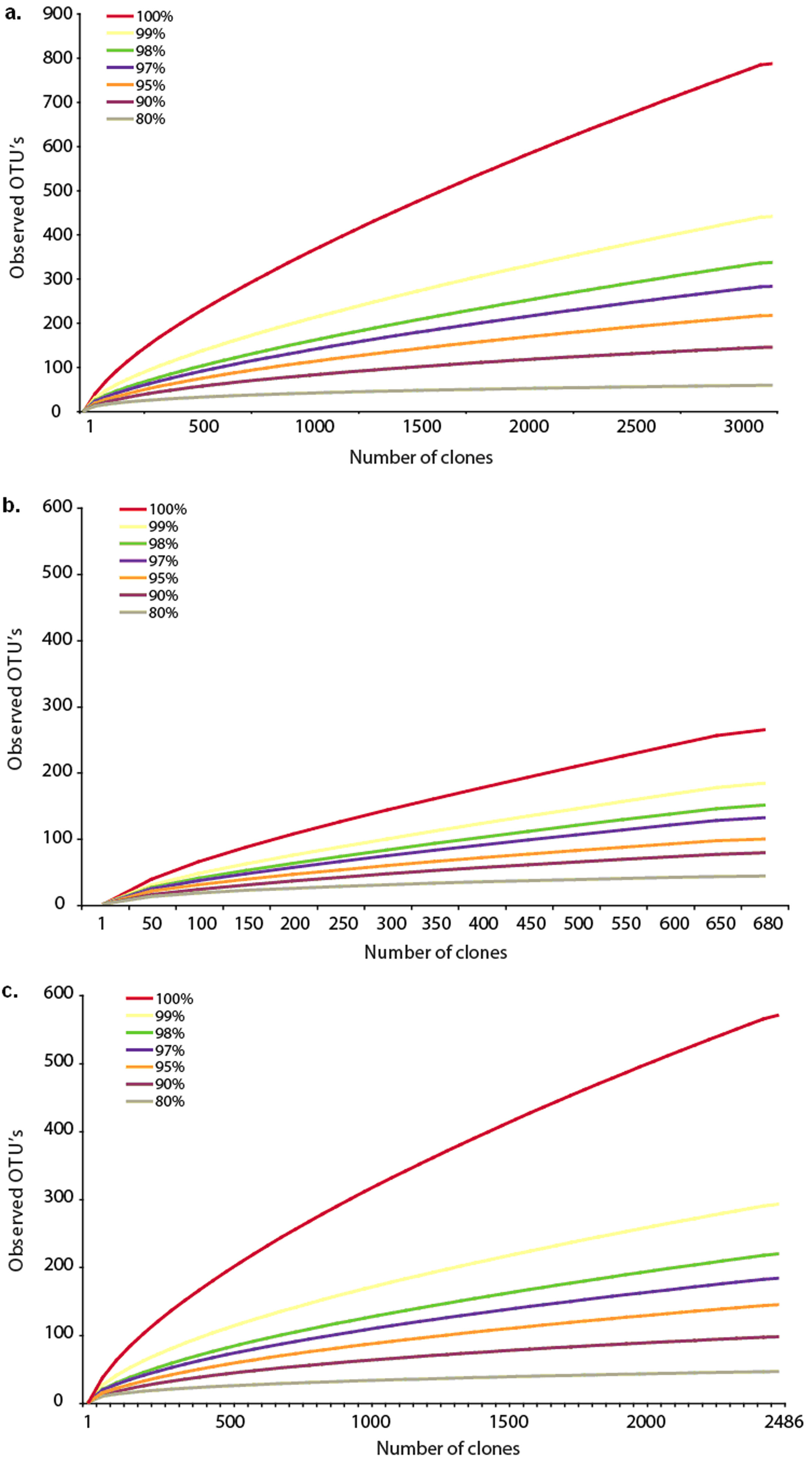

Supplement: Figure S1 — Rarefaction analysis of the leaf-cutter ant fungus garden full-length 16S rDNA sequences. The combined samples (a), top layer samples (b), and bottom layer (c) samples are plotted as shown. Observed Operational Taxonomic Unit (OTUs) cutoffs at 0.00 (100%), 0.01 (99%), 0.02 (98%), 0.03 (97%), 0.05 (95%), 0.10 (90%), and 0.20 (80%) are plotted as a function of the number of clones. (1.72 MB TIF) [file pgen.1001129.s001.tif]

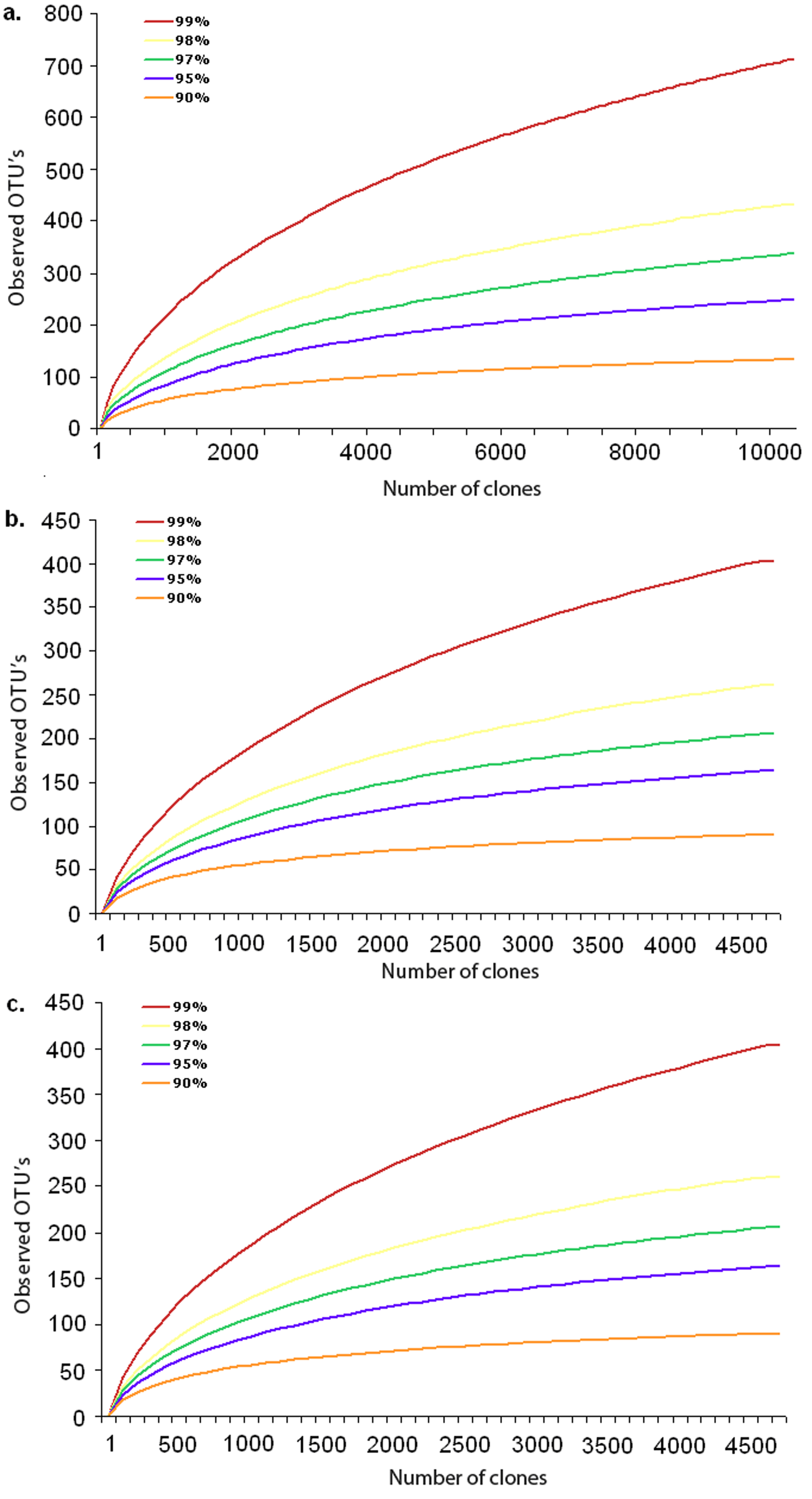

Supplement: Figure S2 — Rarefaction analysis of the leaf-cutter ant fungus garden short-read pyrotagged 16S rDNA sequences. The combined samples (a), top layer samples (b), and bottom layer samples (c) are plotted as shown. Observed Operational Taxonomic Unit (OTUs) cutoffs were determined at 0.01 (99%), 0.02 (98%), 0.03 (97%), 0.05 (95%), and 0.10 (90%) are plotted as a function of the number of clones. (1.14 MB TIF) [file pgen.1001129.s002.tif]

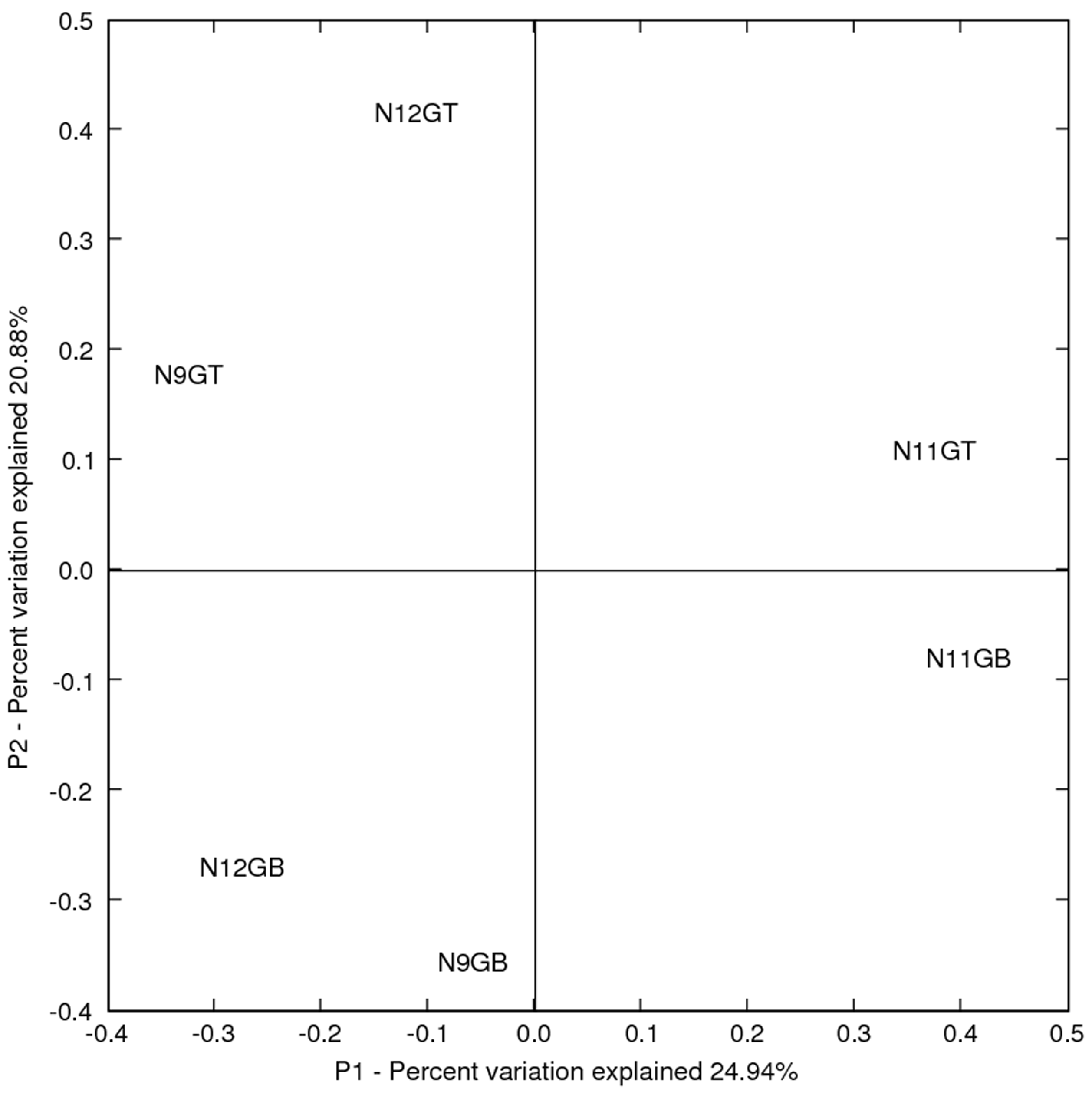

Supplement: Figure S3 — Comparison of the microbial communities from leaf-cutter ant fungus garden top and bottom sample. The plot was generated using unweighted UniFrac. GT = garden top; GB = garden bottom. (0.37 MB TIF) [file pgen.1001129.s003.tif]

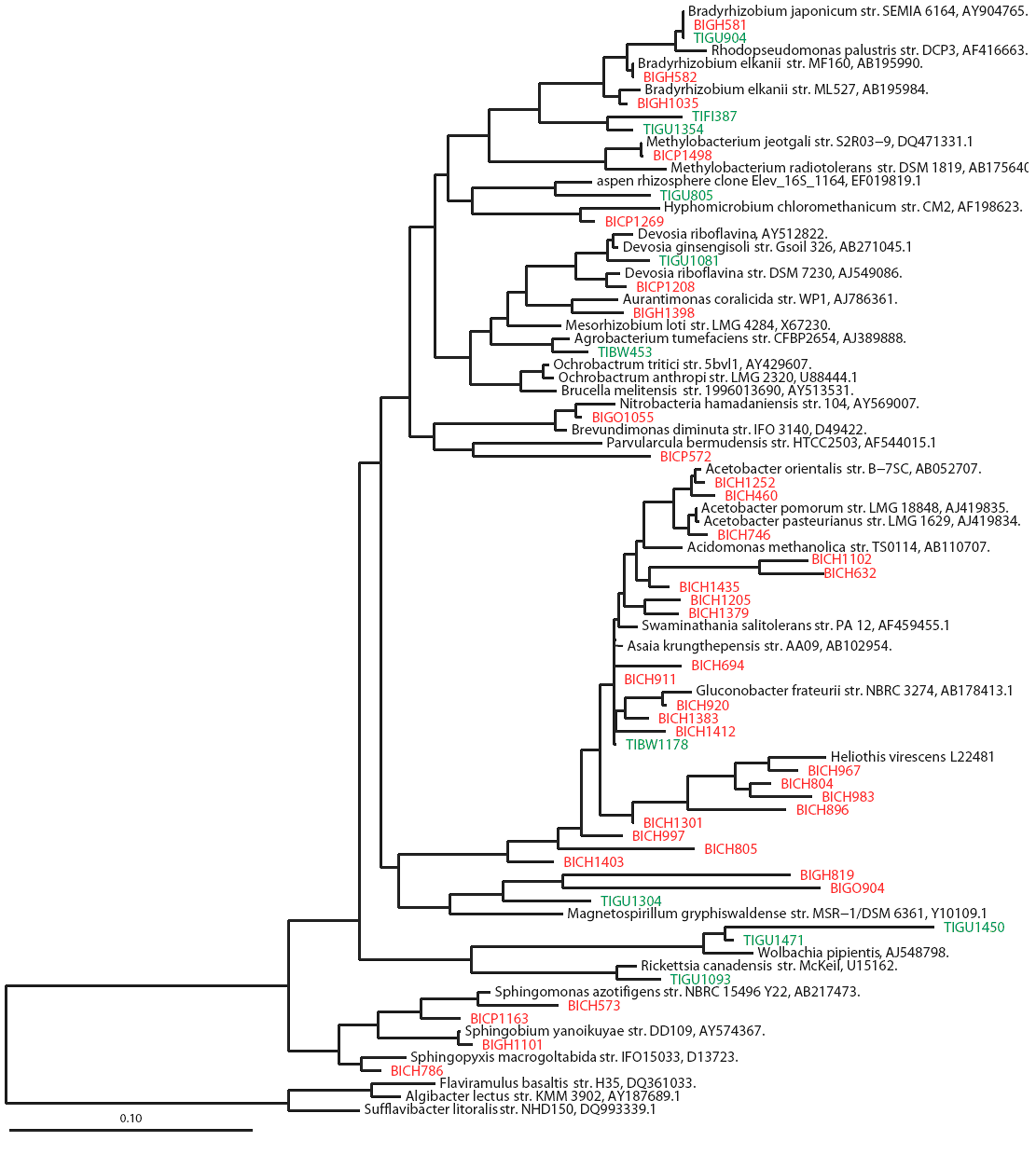

Supplement: Figure S4 — Phylogenetic diversity of α-proteobacteria in the leaf-cutter ant fungus garden near-full length 16S rDNA sequence library. The shown phylogram was constructed using Maximum Likelihood analysis (RAxML) with 11 near-full length 16S rDNA sequences from the garden top (green), 36 sequences from the garden bottom (red), and other closest-matching 16S rDNA sequences from the Greengenes database. GenBank Accession numbers are also provided for Greengene sequences. (2.51 MB TIF) [file pgen.1001129.s004.tif]

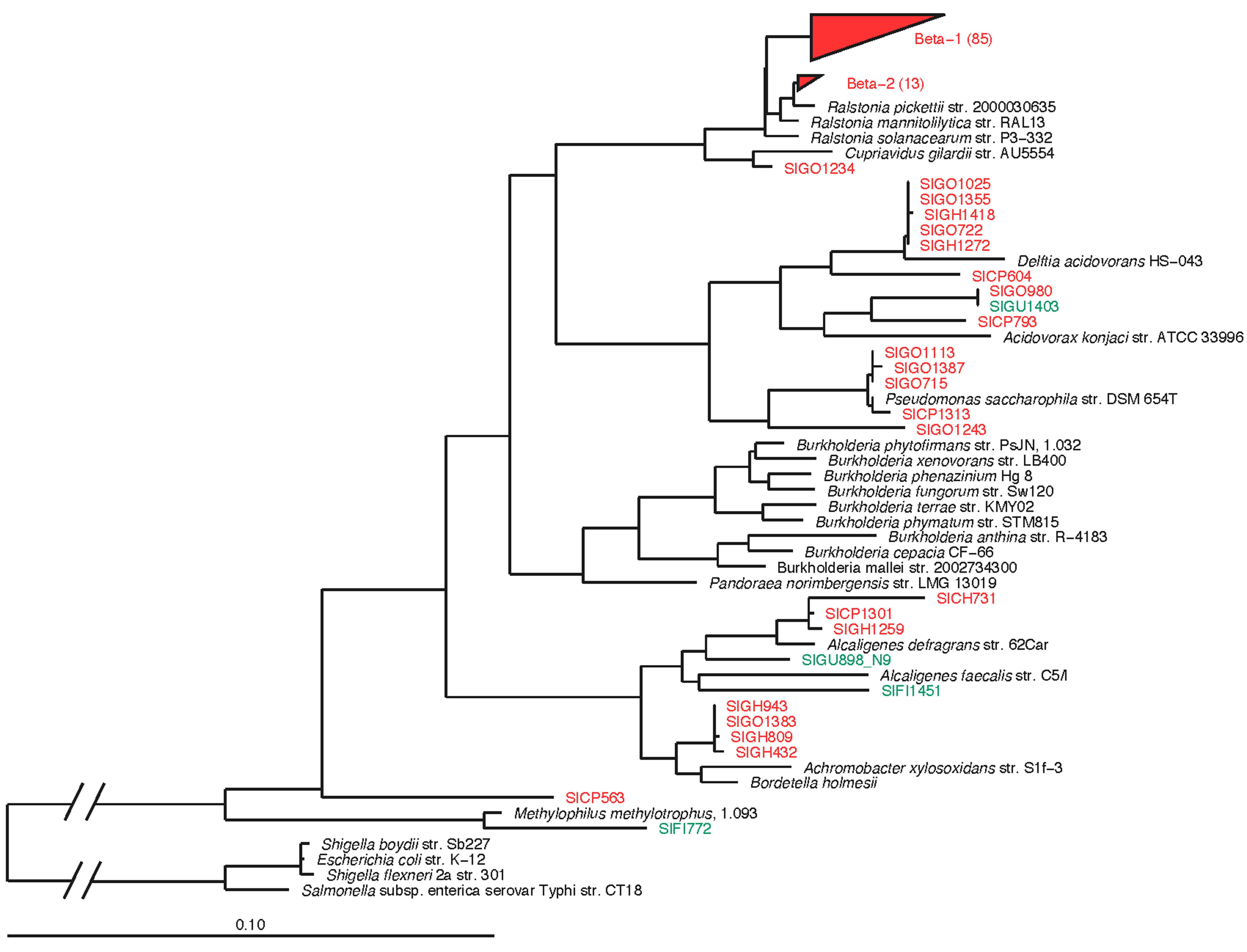

Supplement: Figure S5 — Phylogenetic diversity of β-proteobacteria in the leaf-cutter ant fungus garden near-full length 16S rDNA sequence library. The shown phylogram was constructed using Maximum Likelihood analysis (RAxML) with 4 near-full length 16S rDNA sequences from the garden top (green), 120 sequences from the garden bottom (red), and other closest-matching 16S rDNA sequences from the Greengenes database. GenBank Accession numbers are also provided for Greengenes sequences (1.98 MB TIF) [file pgen.1001129.s005.tif]

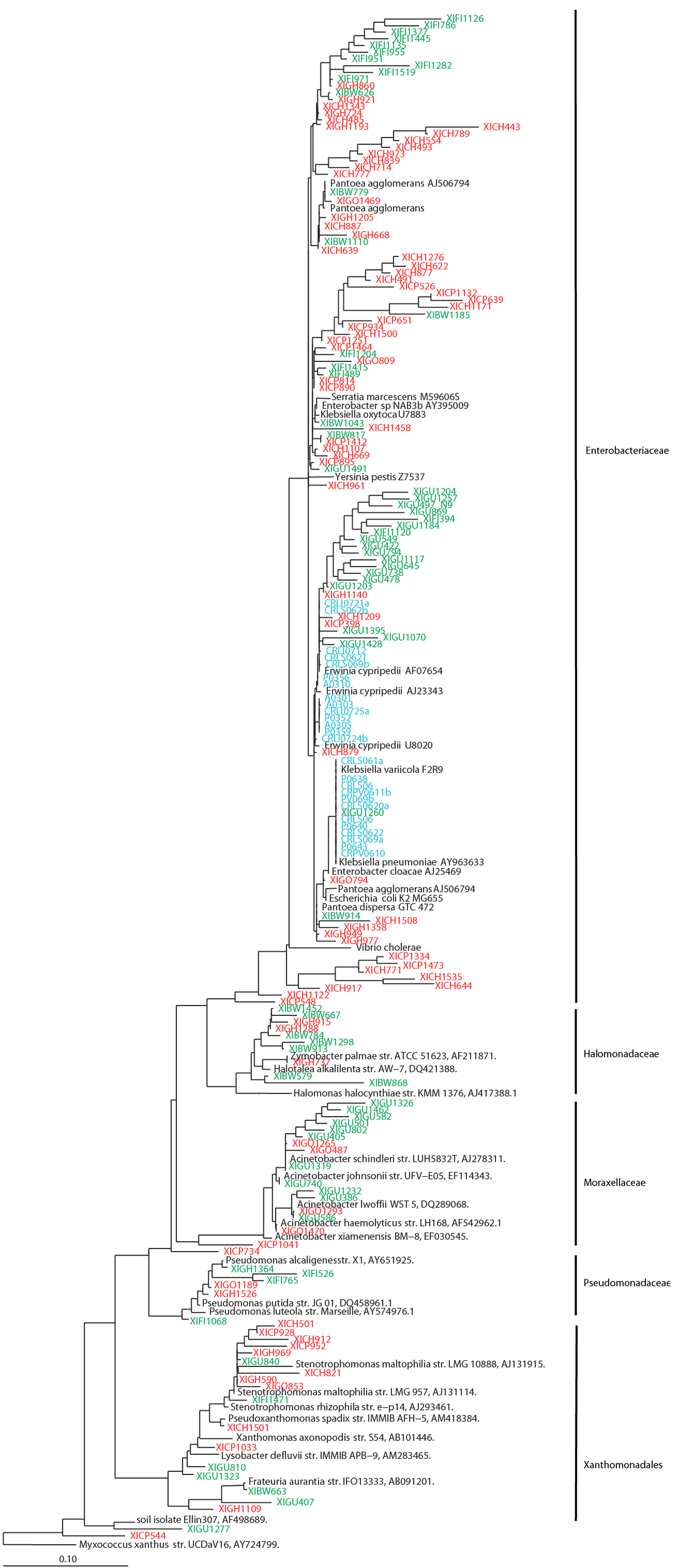

Supplement: Figure S6 — Phylogenetic diversity of γ-proteobacteria in the leaf-cutter ant fungus garden near-full length 16S rDNA sequence library. The shown phylogram was constructed using Maximum Likelihood analysis (RAxML) with 70 near-full length 16S rDNA sequences from the garden top (green), 82 sequences from the garden bottom (red), γ-proteobacterial sequences from previous studies of other leaf-cutter ant fungus gardens (blue), and other closest-matching 16S rDNA sequences from the Greengenes database. GenBank Accession numbers are also provided for Greengene sequences. (6.30 MB TIF) [file pgen.1001129.s006.tif]

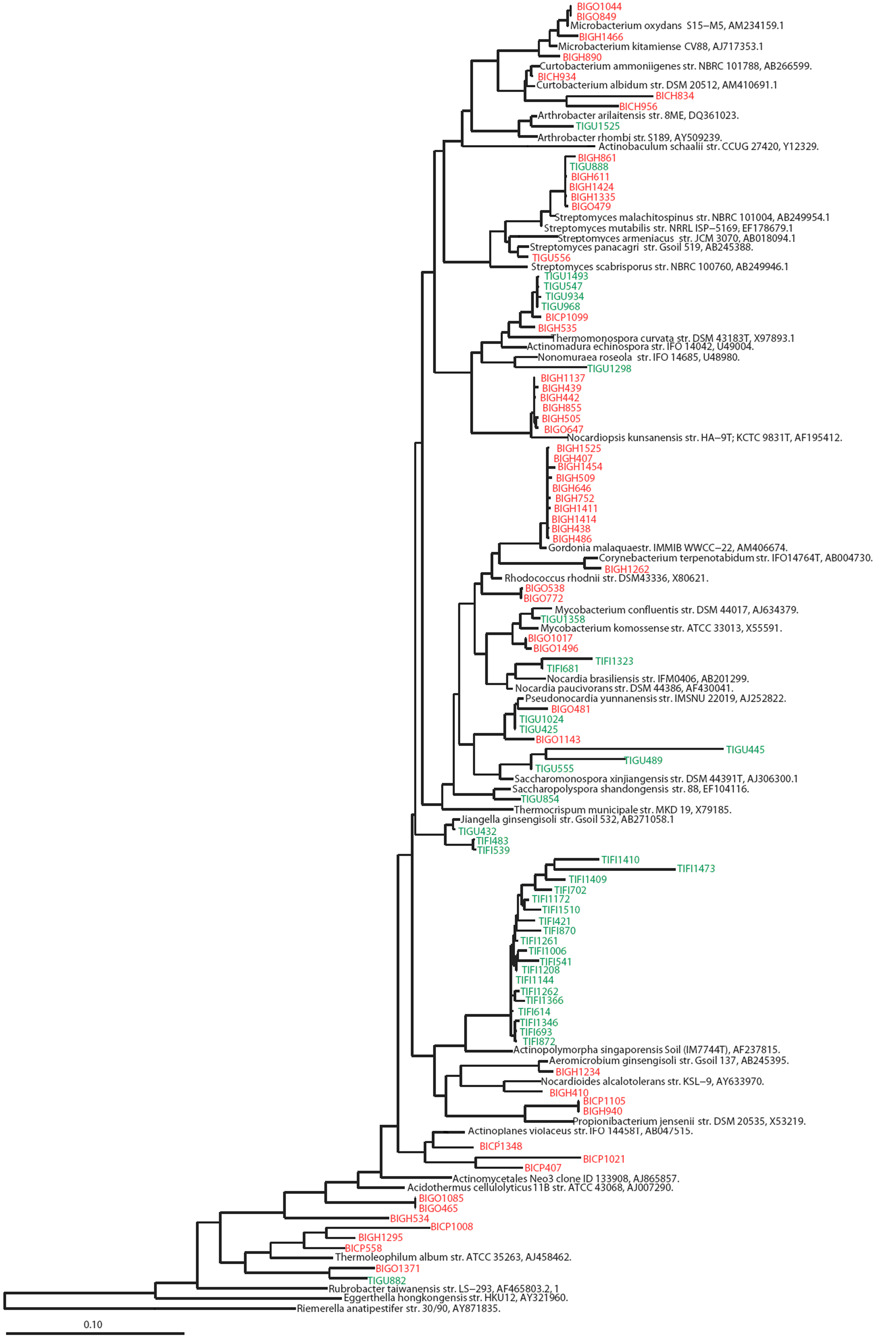

Supplement: Figure S7 — Phylogenetic diversity of Actinobacteria in the leaf-cutter ant fungus garden near-full length 16S rDNA sequence library. The shown phylogram was constructed using Maximum Likelihood analysis (RAxML) with 40 near-full length 16S rDNA sequences from the garden top (green), 51 sequences from the garden bottom (red), and other closest-matching 16S rDNA sequences from the Greengenes database. GenBank Accession numbers are also provided for Greengene sequences. (3.65 MB TIF) [file pgen.1001129.s007.tif]

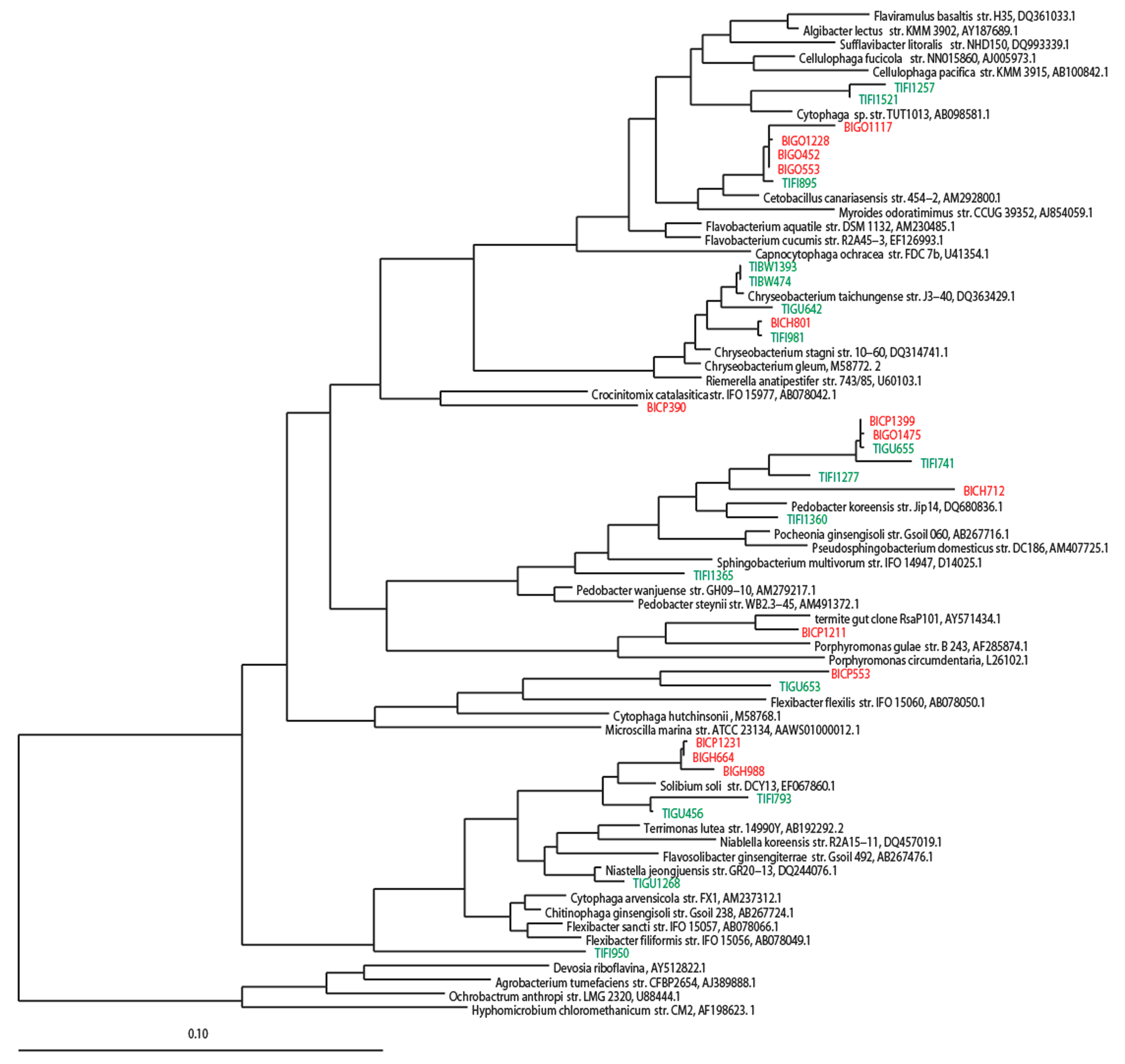

Supplement: Figure S8 — Phylogenetic diversity of Bacteroidetes in the leaf-cutter ant fungus garden near-full length 16S rDNA sequence library. The shown phylogram was constructed using Maximum Likelihood analysis (RAxML) with 17 near-full length 16S rDNA sequences from the garden top (green), 14 sequences from the garden bottom (red), and other closest-matching 16S rDNA sequences from the Greengenes database. GenBank Accession numbers are also provided for Greengene sequences. (2.17 MB TIF) [file pgen.1001129.s008.tif]

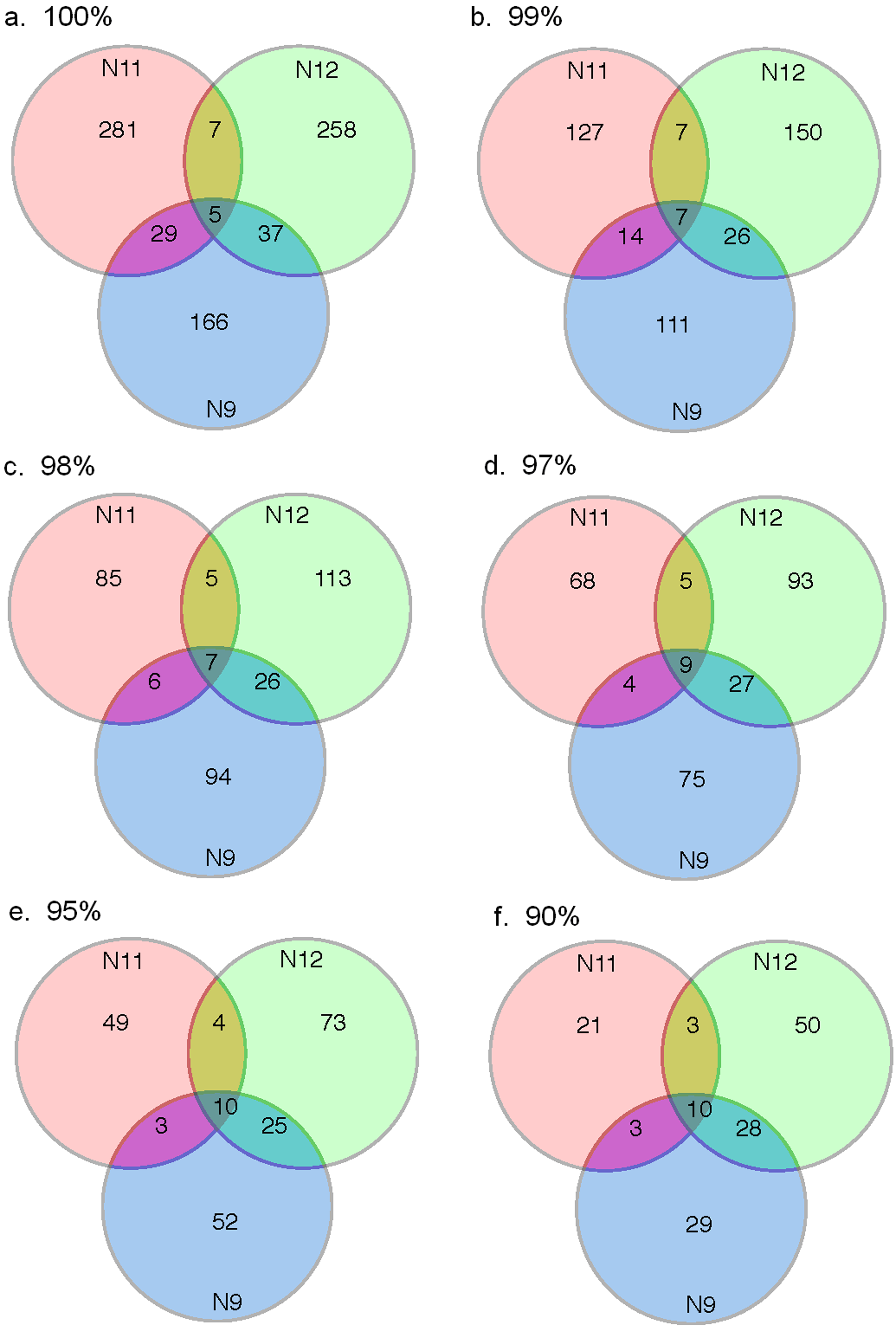

Supplement: Figure S9 — Venn diagram representation of full-length 16S rDNA phylotypes across 3 different colonies of the leaf-cutter ant Atta colombica. Phylotype clusters at different sequence identities are shown at 100% (a), 99% (b), 98% (c), 97% (d), 95% (e), and 90% (f). (2.24 MB TIF) [file pgen.1001129.s009.tif]

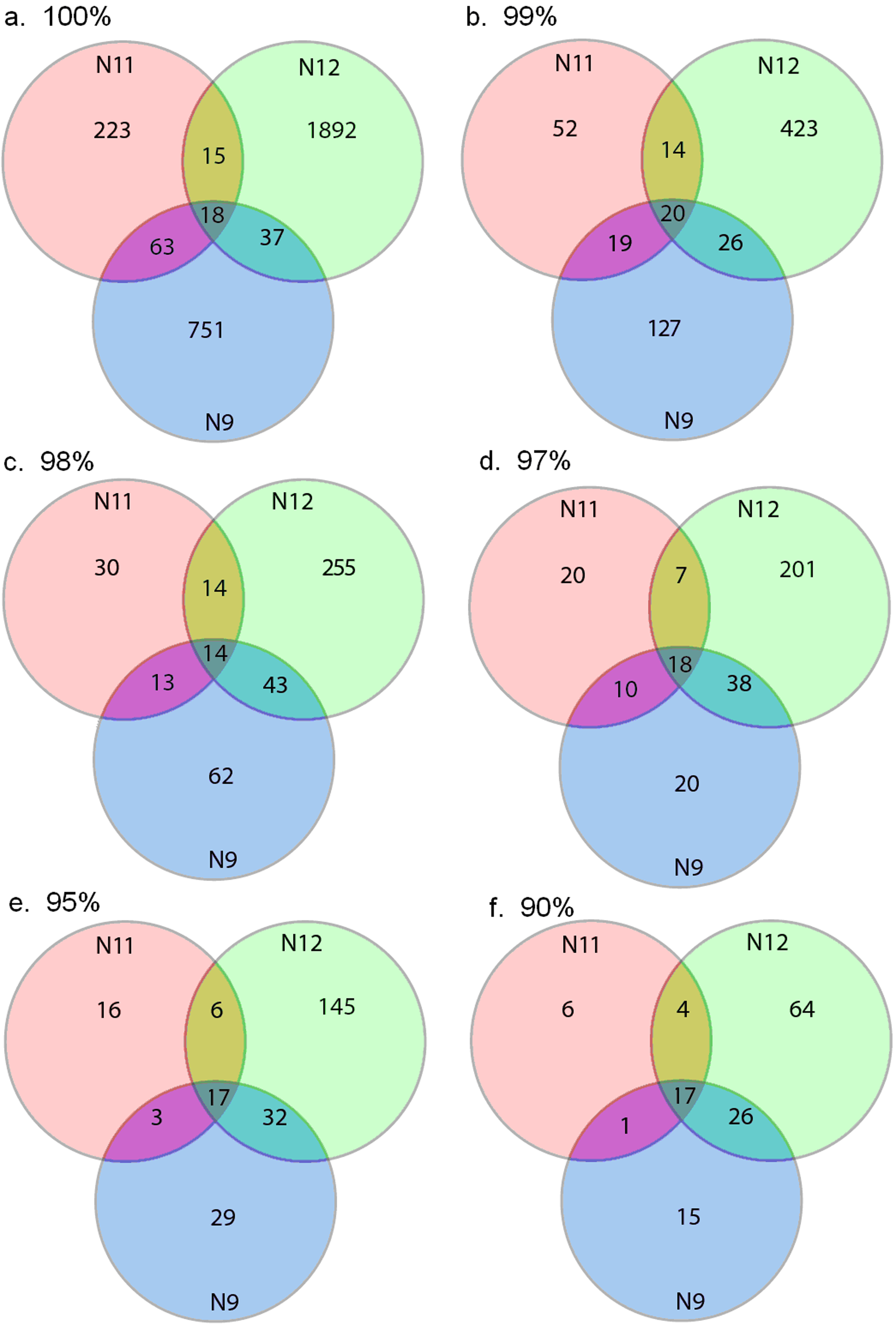

Supplement: Figure S10 — Venn diagram representation of short-read pyrotagged 16S rDNA phylotypes across 3 different colonies of the leaf-cutter ant Atta colombica. Phylotype clusters at different sequence identities are shown at 100% (a), 99% (b), 98% (c), 97% (d), 95% (e), and 90% (f). (2.65 MB TIF) [file pgen.1001129.s010.tif]

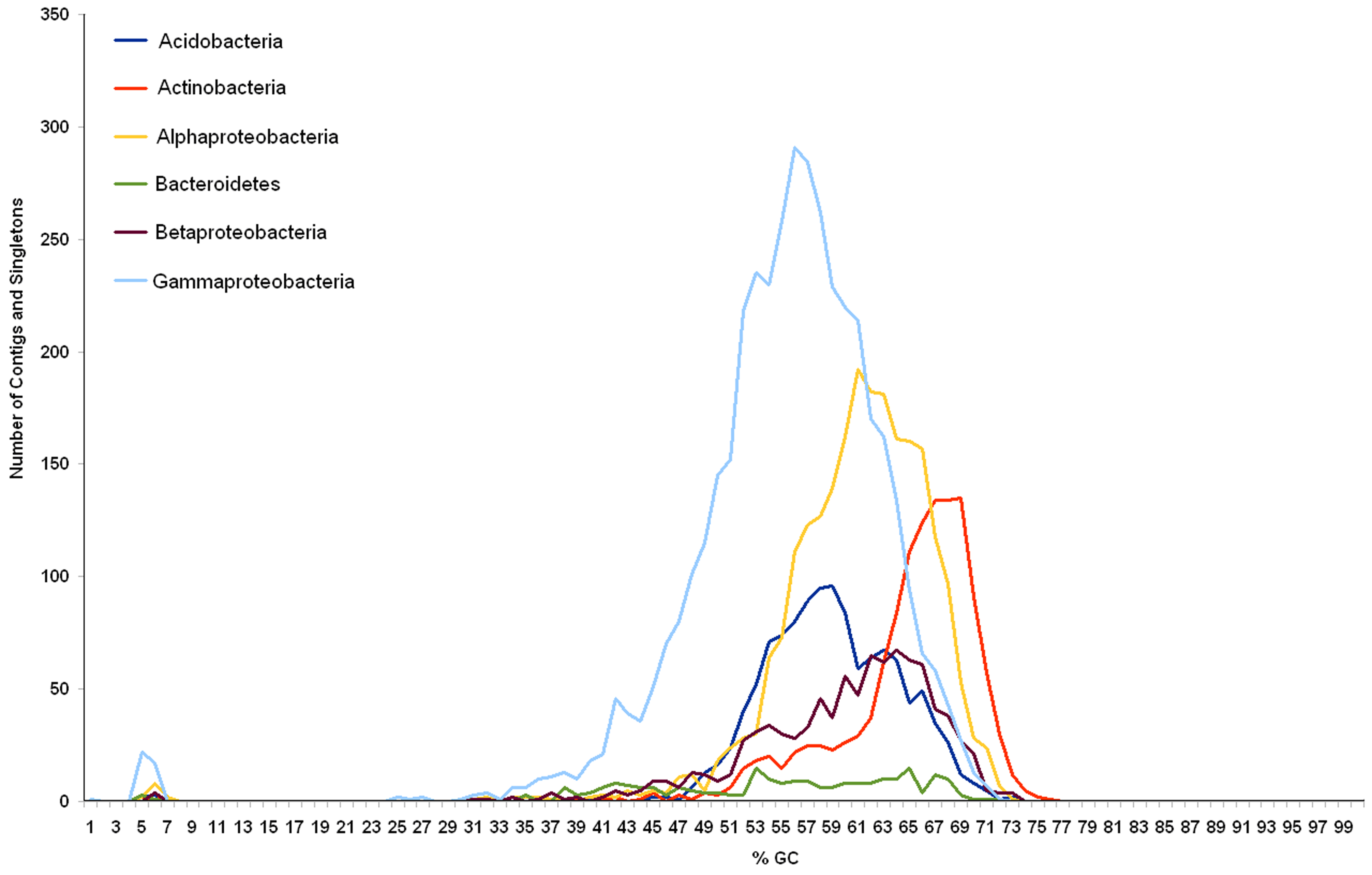

Supplement: Figure S11 — GC content analysis of the bacterial portion of the leaf-cutter ant fungus garden community metagenome. The % GC of each contig and singleton classified as bacterial was tabulated and graphed according to its taxonomic group. The γ-proteobacteria had the highest number of contigs and reads with a % GC commiserate with sequenced γ-proteobacterial genomes. The Actinobacteria had the highest average % GC, as expected based on the average % GC of sequenced Actinobacterial genomes. (0.99 MB TIF) [file pgen.1001129.s011.tif]

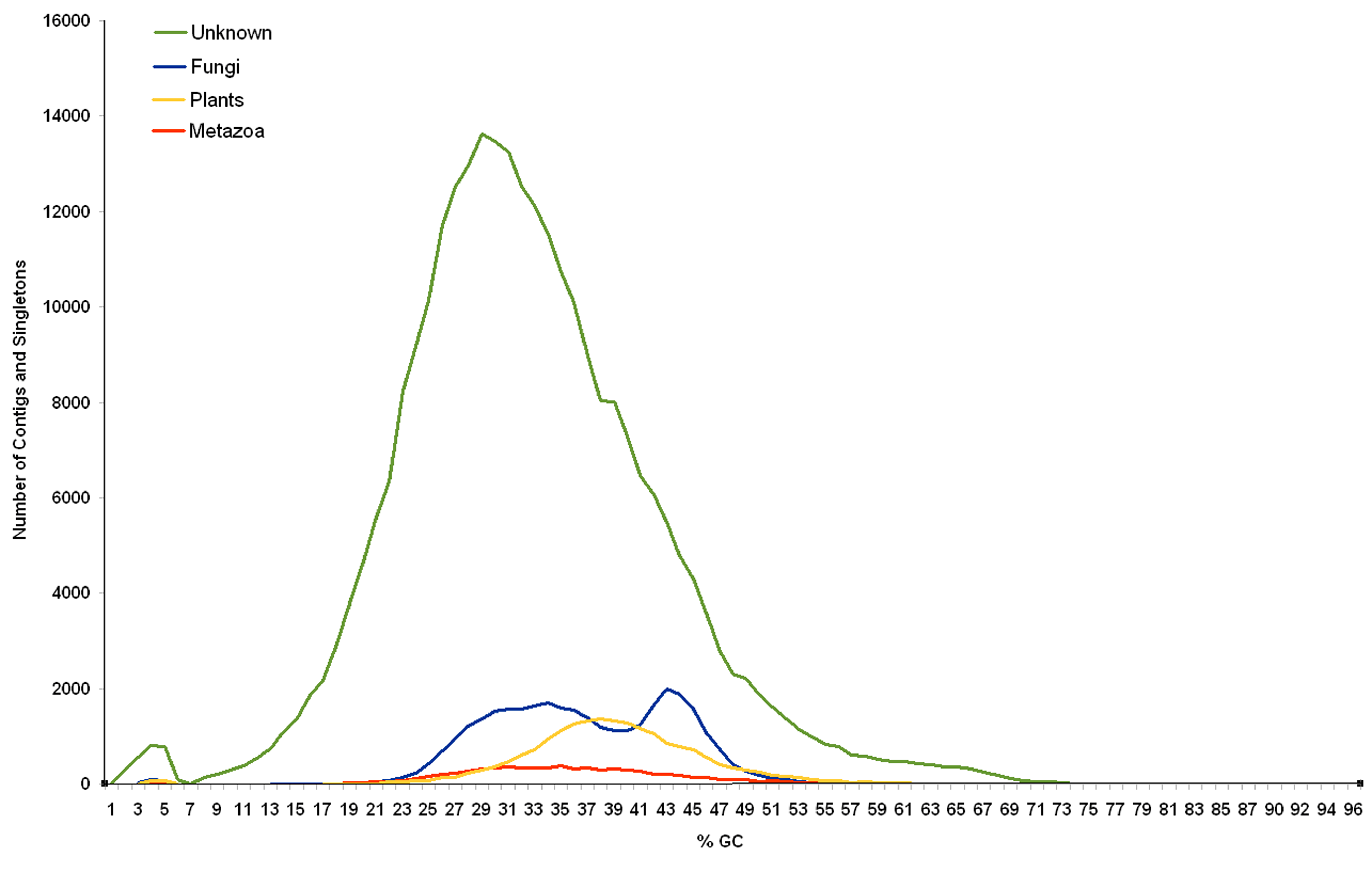

Supplement: Figure S12 — GC content analysis of the eukaryotic and unclassified portion of the leaf-cutter ant fungus garden community metagenome. The % GC of each contig and singleton classified as eukaryotic was tabulated and graphed according to the categories fungi, metazoa, and plants. Calculation of the % GC for the unclassified portion of the leaf-cutter ant fungus garden community metagenome is also shown. (0.70 MB TIF) [file pgen.1001129.s012.tif]

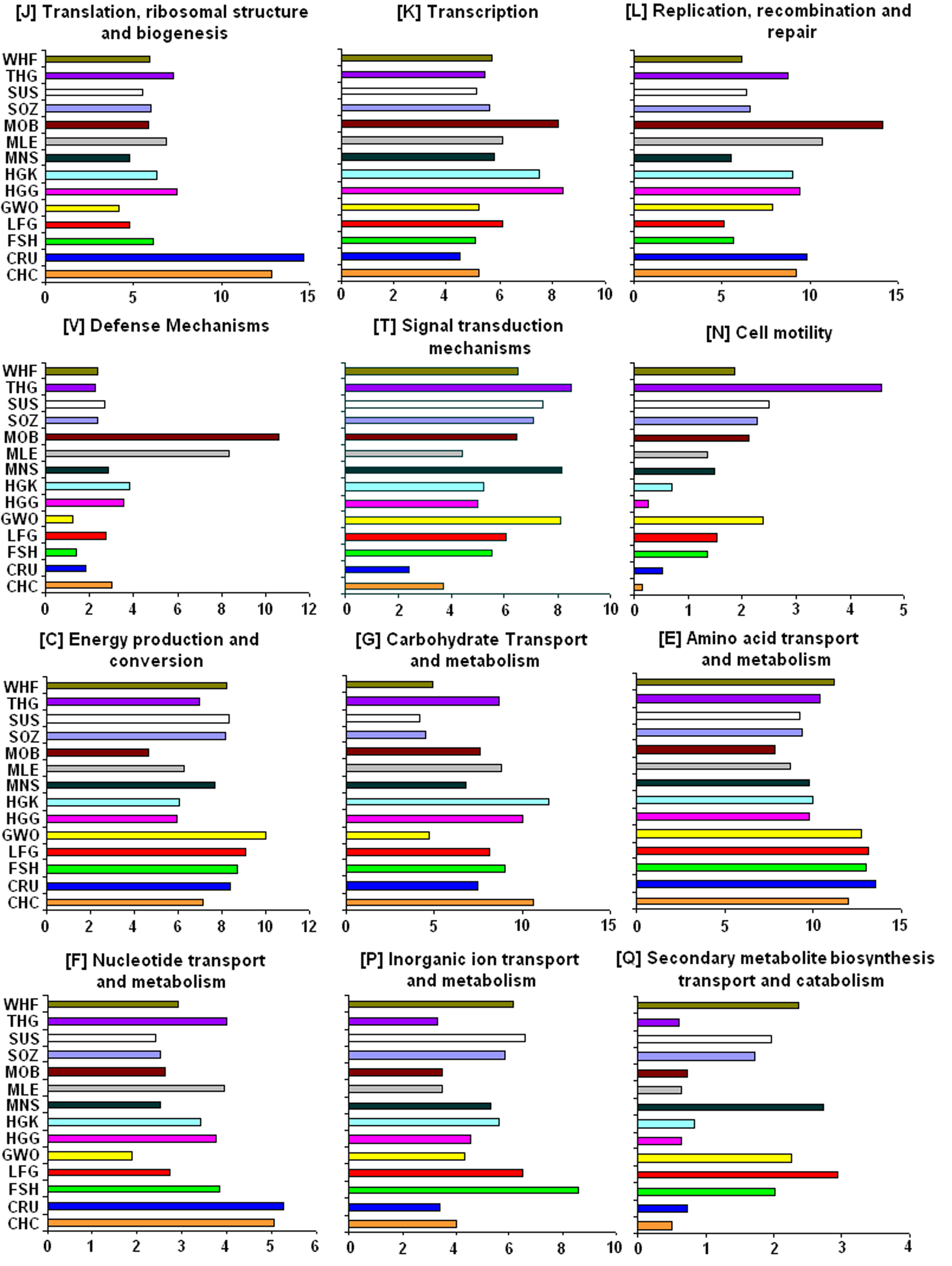

Supplement: Figure S13 — Clusters of orthologous groups (COG) analysis of the leaf-cutter ant fungus garden community metagenome compared to 13 other metagenomes. Shown is the number of COG-annotated proteins in each category, represented as a proportion of each metagenome's total COG-annotated proteins for 12 categories. Abbreviations for each metagenome are as follows: chicken cecum (CHC), cow rumen (CRU), fish (FSH), leaf-cutter ant fungus garden (LFG), gutless worm (GWO), human gut - Gill study (HGG), human gut - Kurokawa study (HGK), Minnesota soil (MNS), mouse lean (MLE), mouse obese (MOB), sludge Australia (SOZ), sludge USA (SUS), termite hindgut (THG), and whale fall (WHF). (1.05 MB TIF) [file pgen.1001129.s013.tif]

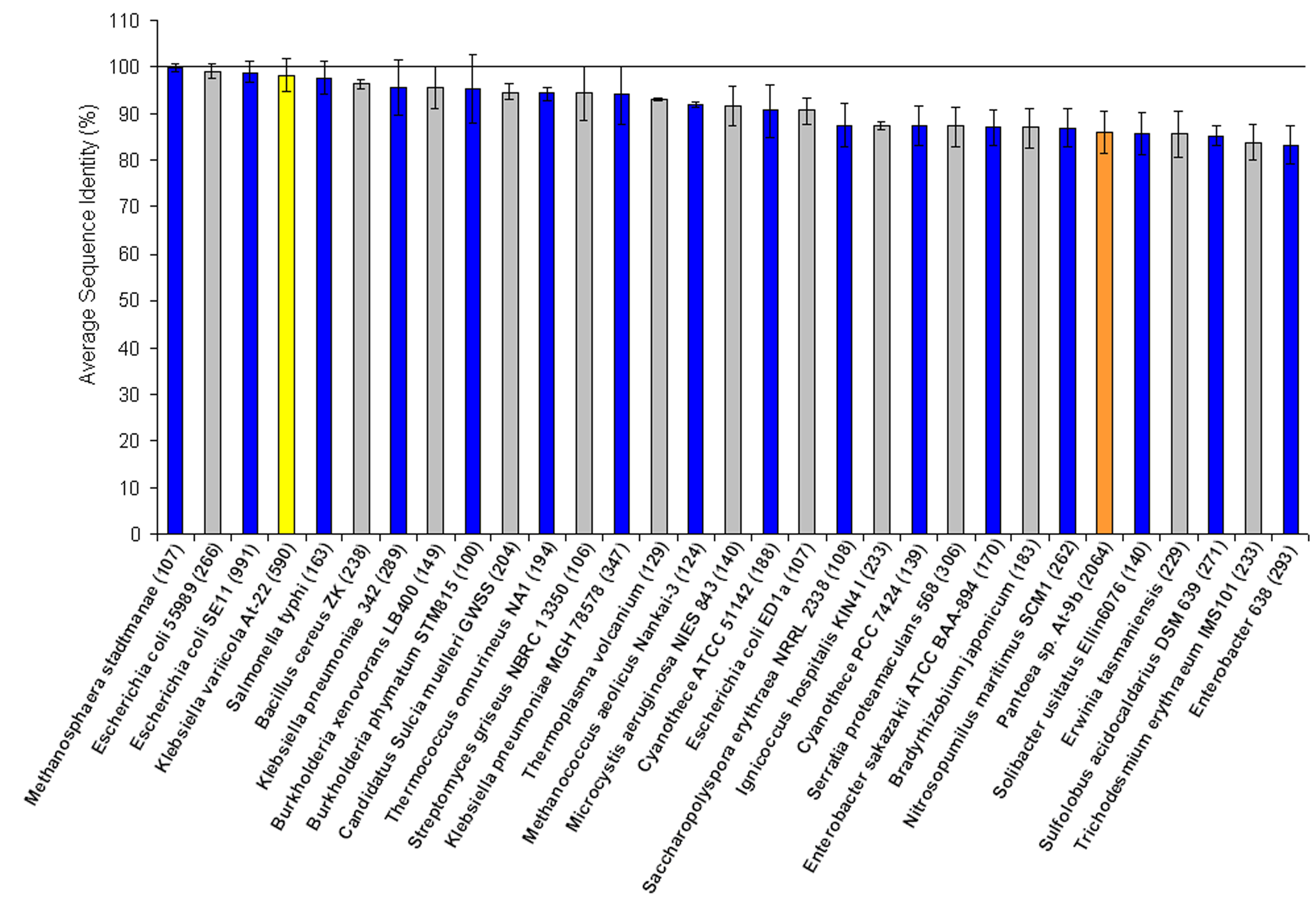

Supplement: Figure S14 — Average sequence identity of leaf-cutter ant fungus garden community metagenome reads mapped onto complete genomes in the microbial genome collection and the draft genomes of the leaf-cutter ant-associated Klebsiella variicola At-22 and Pantoea sp. At-9b. Only those organisms with more than 100 mapped reads are shown. The total number of mapped reads is also listed in parentheses beside each organism's name. Average sequence identities are highlighted for Klebsiella variicola At-22 (yellow) and Pantoea sp. At-9b (orange). Standard deviation bars are also shown. (2.06 MB TIF) [file pgen.1001129.s014.tif]
