# Supplementary material for: An Insect Herbivore Microbiome with High Plant Biomass-Degrading Capacity
Source: PLoS Genet. 2010 Sep 23;6(9):e1001129. doi: 10.1371/journal.pgen.1001129 (PMC2944797; doi:10.1371/journal.pgen.1001129)
Supplement: Table S1 — Summary statistics for near full-length and pyrotag 16S rDNA sequencing of leaf-cutter ant fungus gardens. Sequences were generated for garden top and bottom samples from 3 Atta colombica leaf-cutter ant colonies. Average sequence length and the total number of sequences generated are also shown. (0.03 MB DOC) [file pgen.1001129.s015.doc]

| **Sample** | **No. Full-length 16S rDNA Sequences** | **Average Sequence**  **Length (bp)** | **No. pyrotag**  **16 rDNA Sequences** | **Average Sequence**  **Length (bp)** |
| --- | --- | --- | --- | --- |
| N9 Top | 311 | 1,370 | 111 | 522 |
| N9 Bottom | 610 | 1,255 | 5,699 | 518 |
| N11 Top | 288 | 1,343 | 515 | 527 |
| N11 Bottom | 868 | 1,246 | 657 | 519 |
| N12 Top | 104 | 1,368 | 8,342 | 520 |
| N12 Bottom | 1,316 | 1,208 | 5,006 | 527 |
| **Totals** | **3,497** | **1,256** | **20,330** | **522** |
