# Supplementary material for: An Insect Herbivore Microbiome with High Plant Biomass-Degrading Capacity
Source: PLoS Genet. 2010 Sep 23;6(9):e1001129. doi: 10.1371/journal.pgen.1001129 (PMC2944797; doi:10.1371/journal.pgen.1001129)
Supplement: Table S2 — Summary statistics for the leaf-cutter ant fungus garden community metagenome. Raw sequence reads were generated using 454 titanium pyrosequencing and assembled into contigs using only high-quality reads. Reads that could not be assembled were assigned as singletons. Phylogenetic binning of all contigs and singletons were performed using BLAST and comparing against NCBI's non-redundant nucleotide (nt) database to classify into one of bacterial, eukaryotic, viral, unclassified sets. (0.03 MB DOC) [file pgen.1001129.s016.doc]

| **Characteristic** | **Amount** |
| --- | --- |
| Number of Raw Reads generated | 1,143,425 |
| Raw bases generated | 400,950,367 bp |
| Average read length | 350 bp |
| Assembly Statistics | |
| Number of assembled contigs | 154,661 |
| Largest contig Size | 24,446 bp |
| Average contig Size | 910 bp |
| Total assembled contig length | 67,688,090 bp |
| Number of reads completely or partially assembled | 723,927 |
| Number of singletons | 200,621 |
| Total singleton length | 61,546,920 bp |
| Number of contigs and singletons | 355,282 |
| Total contig and singleton length | 129,235,010 bp |
| Phylogenetic binning against NCBI’s nt | |
| Bacterial Content | 6,156,113 bp |
| Eukaryotic Content | 34,231,593 bp |
| Viral Content | 22,884 bp |
| Unclassified Content | 88,824,420 bp |
