# Supplementary material for: An Insect Herbivore Microbiome with High Plant Biomass-Degrading Capacity
Source: PLoS Genet. 2010 Sep 23;6(9):e1001129. doi: 10.1371/journal.pgen.1001129 (PMC2944797; doi:10.1371/journal.pgen.1001129)
Supplement: Table S3 — Total phylotypes counts for the leaf-cutter ant fungus garden near full-length 16S rDNA library. Phylotypes are at the genus level (97% identity), and classified at the family and taxonomic groups for top, bottom, and combined samples. (0.13 MB DOC) [file pgen.1001129.s017.doc]

| **NCBI Taxonomic Group** | **Family** | **Number of phylotypes** | | |
| --- | --- | --- | --- | --- |
|  |  | **Garden Top** | **Garden Bottom** | **Combined** |
| Acidobacteria | *Acidobacteriaceae* | 6 | 7 | 13 |
| Actinobacteria | *Acidimicrobidae* | 0 | 1 | 1 |
|  | *Actinomycineae* | 0 | 1 | 1 |
|  | *Anaerolineae* | 0 | 1 | 1 |
|  | *Corynebacteriaceae* | 1 | 2 | 3 |
|  | *Microbacteriaceae* | 0 | 7 | 7 |
|  | *Micrococcaceae* | 1 | 0 | 1 |
|  | *Propionibacteriaceae* | 5 | 3 | 8 |
|  | *Pseudonocardiaceae* | 8 | 5 | 13 |
|  | *Rubrobacteridae* | 1 | 4 | 5 |
|  | *Streptomycetaceae* | 2 | 1 | 3 |
|  | *Streptosporangiaceae* | 1 | 4 | 5 |
|  | *Unclassified Actinobacteria family* | 0 | 1 | 1 |
| Bacteroidetes | *Acidaminococcaceae* | 0 | 1 | 1 |
|  | *Bacteroidaceae* | 0 | 1 | 1 |
|  | *Flavobacteriaceae* | 4 | 2 | 6 |
|  | *Flexibacteraceae* | 1 | 1 | 2 |
|  | *Saprospiraceae* | 4 | 1 | 5 |
|  | *Spingobacteriaceae* | 4 | 2 | 6 |
| Chloroflexi | *Ktedobacteria* | 0 | 1 | 1 |
| Firmicutes | *Streptococcaceae* | 0 | 1 | 1 |
|  | *Alicyclobacillaceae* | 0 | 1 | 1 |
|  | *Lactobacillaceae* | 2 | 2 | 4 |
|  | *Paenibacillaceae* | 1 | 0 | 1 |
|  | *Staphylococcaceae* | 0 | 1 | 1 |
| Gemmatimondetes | *Gemmatimondaceae* | 2 | 0 | 2 |
| Nitrospirae | *Nitrospiraceae* | 1 | 0 | 1 |
| Other Bacteria | *-* | 9 | 4 | 13 |
| Planctoymces | *Planctomycetaceae* | 3 | 3 | 6 |
| α-proteobacteria | *Acetobacteraceae* | 1 | 24 | 25 |
|  | *Aurantimonadaceae* | 0 | 1 | 1 |
|  | *Azospirillaceae* | 1 | 0 | 1 |
|  | *Bradyrhizobiaceae* | 1 | 2 | 3 |
|  | *Brucellaceae* | 0 | 1 | 1 |
|  | *Caulobacteraceae* | 0 | 1 | 1 |
|  | *Ceaedibacteraceae* | 0 | 1 | 1 |
|  | *Hyphomicrobiaceae* | 0 | 2 | 2 |
|  | *Methylobacteriacea* | 0 | 1 | 1 |
|  | *Phylobacteriaceae* | 0 | 1 | 1 |
|  | *Rhizobiaceae* | 1 | 0 | 1 |
|  | *Rhodobacteriacea* | 0 | 1 | 1 |
|  | *Rhodoplanaceae* | 2 | 0 | 2 |
|  | *Ricketsiaceae* | 3 | 0 | 3 |
|  | *Sphingonmadaceae* | 0 | 4 | 4 |
|  | *Unclassified α-proteobacteria family* | 2 | 1 | 3 |
| β-proteobacteria | *Alcaligenaceae* | 2 | 3 | 5 |
|  | *Comamonadaceae* | 1 | 6 | 7 |
|  | *Methylophilaceae* | 1 | 0 | 1 |
|  | *Ralstoniaceae* | 0 | 4 | 4 |
|  | *Unclassified β-proteobacteria family* | 0 | 2 | 2 |
| γ-proteobacteria | *Enterobacteriaceae* | 30 | 55 | 85 |
|  | *Halomonadaceae* | 7 | 3 | 10 |
|  | *Moraxellaceae* | 11 | 3 | 14 |
|  | *Pseudomonadaceae* | 3 | 2 | 5 |
|  | *Xanthomonadaceae* | 5 | 8 | 13 |
|  | *Unclassified γ-proteobacteria family* | 0 | 3 | 3 |
| δ-proteobacteria | *Myxococcaceae* | 0 | 1 | 1 |
|  | *Unclassified δ-proteobacteria family* | 2 | 4 | 6 |
| Verrucomicrobia | *Verrucomicrobiaceae* | 3 | 5 | 8 |
| **Total Phylotypes** | | **132** | **197** | **329** |
