# Supplementary material for: An Insect Herbivore Microbiome with High Plant Biomass-Degrading Capacity
Source: PLoS Genet. 2010 Sep 23;6(9):e1001129. doi: 10.1371/journal.pgen.1001129 (PMC2944797; doi:10.1371/journal.pgen.1001129)
Supplement: Table S4 — Total phylotype counts for the leaf-cutter ant fungus garden short-read pyrotagged 16S rDNA library. Phylotypes were determined by sequence comparison against the Greengenes database (97% sequence identity), and tabulated according to NCBI's Taxonomic Group designation. Total phylotypes across all taxonomic groups are displayed for garden top, bottom and the total combined samples. (0.05 MB DOC) [file pgen.1001129.s018.doc]

| **NCBI Taxonomic Group** | **Garden Top** | **Garden Bottom** | **Total** |
| --- | --- | --- | --- |
| Acidobacteria | 33 | 35 | 68 |
| Actinobacteria | 4 | 6 | 10 |
| Bacteroidetes | 22 | 5 | 27 |
| Caldithrix | 0 | 1 | 1 |
| Chloroflexi | 0 | 1 | 1 |
| Firmicutes | 10 | 24 | 34 |
| Gemmatimonadetes | 2 | 0 | 2 |
| NC10 | 0 | 1 | 1 |
| Nitrospirae | 0 | 1 | 1 |
| OP10 | 0 | 1 | 1 |
| Planctomyces | 5 | 3 | 8 |
| α-proteobacteria | 38 | 34 | 72 |
| β-proteobacteria | 25 | 17 | 44 |
| γ-proteobacteria | 36 | 57 | 93 |
| δ-proteobacteria | 10 | 6 | 16 |
| SPAM | 4 | 0 | 4 |
| Thaumarchaeota | 0 | 1 | 1 |
| TM6 | 0 | 1 | 1 |
| Unknown | 2 | 4 | 6 |
| Verrucomicrobia | 2 | 1 | 3 |
| **Total Phylotypes** | **193** | **199** | **394** |
