# Supplementary material for: An Insect Herbivore Microbiome with High Plant Biomass-Degrading Capacity
Source: PLoS Genet. 2010 Sep 23;6(9):e1001129. doi: 10.1371/journal.pgen.1001129 (PMC2944797; doi:10.1371/journal.pgen.1001129)
Supplement: Table S5 — Phylotypes shared across the top and bottom fungus garden layers of three leaf-cutter colonies (N9, N11, and N12). Phylotypes were clustered at a sequence identity of 97% and four comparisons are shown: N11-N12-N9, N11-N12, N11-N9, and N12-N9. A representative clone from each phylotype cluster was used to determine its classification using the type strain collection in the Ribosomal Database Project (RDP). The length of each representative clone, its RDP classification (Genbank identifier in parenthesis) and its RDP sequence identity score are also shown. (0.10 MB DOC) [file pgen.1001129.s019.doc]

| **GARDEN TOP** | | | | |
| --- | --- | --- | --- | --- |
| **Clone ID** | **Comparison** | **Length** | **RDP Classification** | **Identity** |
| SIGU1082 | N11-N12-N9 | 1392 | *Enterobacter cancerogenus*^T^ (Z96078) | 99% |
| SIGU1140 | N11-N12-N9 | 1392 | *Pantoea dispersa*^T^ (DQ504305) | 100% |
| SIBW521 | N11-N12-N9 | 1389 | *Enterobacter hormaechei*^T^ (AJ508302) | 98% |
| SIBW1350 | N11-N12 | 1386 | *Pseudomonas flectens*^T^ (AB021400) | 99% |
| SIBW562 | N11-N9 | 1388 | *Carnimonas nigrificans*^T^ (Y13299) | 97% |
| SIGU888 | N12-N9 | 1375 | *Streptomyces tendae*^T^ (D63873) | 99% |
| SIGU1275 | N12-N9 | 1356 | *Anaplasma phagocytophilum*^T^ (U02521) | 90% |
| SIFI871 | N12-N9 | 1385 | *Enterobacter asburiae*^T^ (AB004744) | 100% |
|  | | | | |
| **GARDEN BOTTOM** | | | | |
| **Clone ID** | **Comparison** | **Length** | **Top BLAST Hit** | **Identity** |
| SIGH1215 | N11-N12-N9 | 1377 | *Pantoea stewartii*^T^ (U80208) | 98% |
| SIGH942 | N11-N12-N9 | 1379 | *Pseudomonas beteli*^T^ (AB021406) | 100% |
| SICH1013 | N11-N12 | 1321 | *Asaia bogorensis*^T^ (AB025928) | 99% |
| SICH1438 | N11-N12 | 1359 | *Tatumella ptyseos*^T^ (AJ233437) | 98% |
| SICP1123 | N11-N12 | 1374 | *Enterobacter hormaechei*^T^ (AJ508302) | 99% |
| SICP651 | N11-N12 | 1324 | *Enterobacter asburiae*^T^ (AB004744) | 93% |
| SICP728 | N11-N12 | 1374 | *Pantoea ananatis*^T^ (U80196) | 100% |
| SICH1014 | N11-N9 | 1372 | *Pantoea citrea*^T^ (DQ838096) | 99% |
| SICH572 | N11-N9 | 1372 | *Pantoea dispersa*^T^ (DQ504305) | 99% |
| SICH888 | N11-N9 | 1372 | *Kluyvera cryocrescens*^T^ (AF310218) | 99% |
| SICP1105 | N12-N9 | 1357 | *Propionibacterium acnes*^T^ (AB042288) | 100% |
| SICP1301 | N12-N9 | 1376 | *Castellaniella denitrificans*^T^ (U82826) | 100% |
| SICP674 | N12-N9 | 1349 | *Enterobacter cancerogenus*^T^ (Z96078) | 95% |
| SICP762 | N12-N9 | 1372 | *Ralstonia insidiosa*^T^ (AF488779) | 100% |
| SIGH1035 | N12-N9 | 1324 | *Bradyrhizobium pachyrhizi*^T^ (AY624135) | 97% |
| SIGH1137 | N12-N9 | 1363 | *Nocardiopsis alba*^T^ (X97883) | 99% |
| SIGH1169 | N12-N9 | 1297 | *Brevundimonas intermedia*^T^ (AJ227786) | 98% |
| SIGH1242 | N12-N9 | 1367 | *Pectobacterium cypripedii*^T^ (AJ233413) | 99% |
| SIGH1272 | N12-N9 | 1367 | *Delftia tsuruhatensis*^T^ (AB075017) | 100% |
| SIGH1305 | N12-N9 | 1320 | *Bradyrhizobium jicamae*^T^ (AY624134) | 100% |
| SIGH432 | N12-N9 | 1368 | *Bordetella petrii*^T^ (AJ249861) | 99% |
| SIGH496 | N12-N9 | 1319 | *Ochrobactrum cytisi*^T^ (AY776289) | 100% |
| SIGH564 | N12-N9 | 1375 | *Escherichia fergusonii*^T^ (AF530475) | 99% |
| SIGH581 | N12-N9 | 1322 | *Bradyrhizobium betae*^T^ (AY372184) | 99% |
| SIGH664 | N12-N9 | 1379 | *Sediminibacterium salmoneum*^T^ (EF407879) | 95% |
| SIGO1475 | N12-N9 | 1374 | *Sphingobacterium multivorum*^T^ (AB100738) | 92% |
| SIGO697 | N12-N9 | 1375 | *Erwinia amylovora*^T^ (AJ233410) | 98% |
