# Supplementary material for: An Insect Herbivore Microbiome with High Plant Biomass-Degrading Capacity
Source: PLoS Genet. 2010 Sep 23;6(9):e1001129. doi: 10.1371/journal.pgen.1001129 (PMC2944797; doi:10.1371/journal.pgen.1001129)
Supplement: Table S6 — Comparison of the top 25 phylogenetic ranks as determined using either the contigs/singletons or reads from the leaf-cutter ant fungus garden metagenome. For reference, binning of the metagenome (contigs/singletons) against the complete microbial genome collection is shown. The rank of each phylogenetic bin and its corresponding nucleotide count is shown. (0.07 MB DOC) [file pgen.1001129.s020.doc]

| **Genus** | **Taxonomic Group** | **Contigs/Singletons vs. Genome collection (nucleotide)** | **Reads vs.**  **Genome collection (nucleotide)** | **Reads vs. Non-redundant**  **nucelotide database** |
| --- | --- | --- | --- | --- |
| *Pantoea* | γ-proteobacteria | 1 (535,392) | 1 (827,495) | 98 (14,029) |
| *Klebsiella* | γ -proteobacteria | 2 (286,032) | 3 (478,156) | 1 (302,430) |
| *Bradyrhizobium* | α-proteobacteria | 3 (109,462) | 8 (144,654) | 6 (148,744) |
| *Serratia* | γ -proteobacteria | 4 (81,025) | 10 (118,873) | 7 (144,016) |
| *Methylobacterium* | α-proteobacteria | 5 (71,411) | 17 (96,781) | 11 (113,893) |
| *Rhodopseudomonas* | α-proteobacteria | 6 (70,871) | 13 (116,316) | 23 (63,224) |
| *Streptomyces* | Actinobacteria | 7 (69,344) | 20 (86,938) | 8 (131,288) |
| *Pseudomonas* | γ -proteobacteria | 8 (63,984) | 7 (150,945) | 13 (113,557) |
| *Burkholderia* | β-proteobacteria | 9 (65,098) | 5 (173,658) | 9 (133,763) |
| *Enterobacter* | γ -proteobacteria | 10 (72,117) | 6 (174,807) | 5 (203,965) |
| *Anaeromyxobacter* | δ-proteobacteria | 11 (54,832) | 23 (82,073) | 18 (76,825) |
| *Solibacter* | Acidobacteria | 12 (47,848) | 25 (62,350) | 3 (295,430) |
| *Erwinia* | γ -proteobacteria | 13 (39,935) | 18 (86,666) | 2 (291,995) |
| *Mycobacterium* | Actinobacteria | 14 (42,108) | 24 (59,999) | 17 (79,463) |
| *Rhizobium* | α-proteobacteria | 15 (36,392) | 32 (50,103) | 27 (47,112) |
| *Salmonella* | γ -proteobacteria | 16 (35,192) | 11 (116,077) | 16 (89,536) |
| *Escherichia* | γ -proteobacteria | 17 (52,529) | 2 (732,678) | 10 (130,332) |
| *Frankia* | Actinobacteria | 18 (34,092) | 37 (45,172) | 22 (66,030) |
| *Acidobacteria* | Acidobacteria | 19 (32,107) | 40 (42,984) | 4 (211,456) |
| *Ralstonia* | β-proteobacteria | 20 (30,259) | 35 (42,952) | 24 (57,250) |
| *Saccharopolyspora* | Actinobacteria | 21 (29,082) | 33 (43,155) | 44 (35,123) |
| *Roseiflexus* | Chloroflexi | 22 (29,067) | 41 (40,594) | 15 (105,824) |
| *Sorangium* | δ-proteobacteria | 23 (27,235) | 44 (37,973) | 21 (64,548) |
| *Gluconobacter* | α-proteobacteria | 24 (26,348) | 49 (31,057) | 25 (52,643) |
| *Rhodococcus* | Actinobacteria | 25 (22,675) | 47 (32,248) | 47 (32,454) |
