# Supplementary material for: An Insect Herbivore Microbiome with High Plant Biomass-Degrading Capacity
Source: PLoS Genet. 2010 Sep 23;6(9):e1001129. doi: 10.1371/journal.pgen.1001129 (PMC2944797; doi:10.1371/journal.pgen.1001129)
Supplement: Table S7 — Represented microbial taxonomic groups in the leaf-cutter ant fungus garden community metagenome. The bacterial portion of the fungus garden metagenome was compared against NCBI's non-redundant nucleotide (nr) database and the total amount of sequence corresponding to each taxonomic group was retained and shown. The percentage of each taxonomic group's represented sequence in the total bacterial portion of the fungus garden community metagenome is also shown. A second phylogenetic binning using the computer program PhymmBL was also performed and produced similar results as shown. (0.05 MB DOC) [file pgen.1001129.s021.doc]

| **NCBI Taxonomic Group** | **Non-Redundant Nucleotide**  **Represented Sequences (bp)** | **PhymmBL**  **Represented Sequences (bp)** |
| --- | --- | --- |
| Acidobacteria | 299,977 (4.87%) | 11,575 (0.19%) |
| Actinobacteria | 552,104 (8.96%) | 842,532 (13.67%) |
| α-proteobacteria | 994,554 (16.14%) | 1,480,822 (24.03%) |
| Bacteroidetes/Chlorobi | 117,710 (1.91%) | 68,529 (1.11%) |
| β-proteobacteria | 411,107 (6.67%) | 883,323 (14.34%) |
| Chlamydiae/Verrucomicrobia | 350 (0.01%) | 210 (0%) |
| Chloroflexi | 236,150 (3.83%) | 49,749 (0.81%) |
| Crenarchaeota | 17,039 (0.28%) | 281 (0%) |
| Cyanobacteria | 222,743 (3.62%) | 38,492 (0.62%) |
| Deinococcus-Thermus | 20,808 (0.34%) | 29,587 (0.48%) |
| δ-proteobacteria | 447,130 (7.26%) | 144,611 (2.35%) |
| ε-proteobacteria | 3,667 (0.06%) | 193 (0%) |
| Euryarchaeota | 49,843 (0.81%) | 82,941 (1.35%) |
| Firmicutes | 181,201 (2.94%) | 198,567 (3.22%) |
| γ-proteobacteria | 1,818,055 (29.51%) | 2,096,759 (34.03%) |
| Gemmatimonadetes | 7,906 (0.13%) | 7,680 (0.12%) |
| Other Bacteria | 545,343 (8.85%) | 168,163 (2.73%) |
| Planctomycetes | 80,053 (1.3%) | 18,127 (0.29%) |
| Spirochaetes | 5,888 (0.1%) | 3,526 (0.06%) |
| Thermotogae | 13,150 (0.21%) | 2,438 (0.04%) |
| Verrucomicrobia | 136,783 (2.22%) | 33,456 (0.54%) |
| **Total Sequences** | **6,161,561** | **6,161,561** |
