# Supplementary material for: An Insect Herbivore Microbiome with High Plant Biomass-Degrading Capacity
Source: PLoS Genet. 2010 Sep 23;6(9):e1001129. doi: 10.1371/journal.pgen.1001129 (PMC2944797; doi:10.1371/journal.pgen.1001129)
Supplement: Table S8 — Top 20 eukaryotic phylogenetic bins of the leaf-cutter ant fungus garden metagenome as determined by comparison against NCBI's non-redundant nucleotide database (nt). Ranks are determined by the highest total nucleotide coverage at the genus level (Shown in parenthesis after each taxa). The classification designation for each genus is also shown. (0.04 MB DOC) [file pgen.1001129.s022.doc]

| **Genus** | **Classification** | **Non-Redundant**  **Nucleotide Binning** |
| --- | --- | --- |
| *Coprinopsis* | Fungi | 1 (8,392,009) |
| *Laccaria* | Fungi | 2 (6,609,545) |
| *Tricholoma* | Fungi | 3 (3,787,858) |
| *Vitis* | Plant | 4 (3,583,980) |
| *Moniliophthora* | Fungi | 5 (1,919,775) |
| *Apis* | Insect | 6 (1,397,889) |
| *Oryza* | Plant | 7 (1,120,097) |
| *Arabidopsis* | Plant | 8 (1,058,814) |
| *Nasonia* | Insect | 9 (616,804) |
| *Postia* | Fungi | 10 (574,090) |
| *Medicago* | Plant | 11 (249,610) |
| *Solanum* | Plant | 12 (225,586) |
| *Populus* | Plant | 13 (216,827) |
| *Cryptococcus* | Fungi | 14 (196,080) |
| *Pisum* | Plant | 15 (177,488) |
| *Petunia* | Plant | 16 (146,138) |
| *Agaricus* | Fungus | 17 (145,468) |
| *Aspergillus* | Fungus | 18 (119,745) |
| *Drosophila* | Insect | 19 (115,648) |
| *Tribolium* | Insect | 20 (103,693) |
