# Supplementary material for: An Insect Herbivore Microbiome with High Plant Biomass-Degrading Capacity
Source: PLoS Genet. 2010 Sep 23;6(9):e1001129. doi: 10.1371/journal.pgen.1001129 (PMC2944797; doi:10.1371/journal.pgen.1001129)
Supplement: Table S9 — Comparison of the leaf-cutter ant fungus garden metagenome against those of 13 other metagenome using carbohydrate-active enzyme (CAZy) profiles. Shown is the total proportion of CAZy-annotated enzymes (confirmed by Pfam), by family, in each metagenome's predicted CAZyme. Abbreviations are as follows: chicken cecum (CHC), cow rumen (CRU), fish (FSH), leaf-cutter ant fungus garden (LFG), gutless worm (GWO), human gut - Gill study (HGG), human gut - Kurokawa study (HGK), Minnesota soil (MNS), mouse lean (MLE), mouse obese (MOB), sludge Australia (SOZ), sludge USA (SUS), termite hindgut (THG), and whale fall (WHF). (0.21 MB DOC) [file pgen.1001129.s023.doc]

| **CAZy** | **CHC** | **CRU** | **FSH** | **LFG** | **GWO** | **HGG** | **HGK** | **MNS** | **MLE** | **MOB** | **SOZ** | **SUS** | **THG** | **WHF** |
| --- | --- | --- | --- | --- | --- | --- | --- | --- | --- | --- | --- | --- | --- | --- |
| CBM2 | - | - | - | - | - | - | - | - | - | - | 1.37 | - | - | - |
| CBM3 | - | - | - | - | - | - | 0.05 | - | - | - | - | - | - | - |
| CBM32 | - | - | - | - | - | - | 5.56 | 1.29 | - | - | 1.37 | - | - | - |
| CBM33 | - | - | - | - | - | - | 0.05 | 0.22 | - | - | - | - | - | - |
| CBM34 | - | - | - | - | - | 0.93 | 0.20 | - | - | - | - | - | - | - |
| CBM4 | - | - | - | - | - | 0.93 | 0.10 | - | - | - | - | - | - | 0.36 |
| CBM40 | - | - | - | - | - | 0.47 | 0.10 | - | - | - | - | - | - | - |
| CBM5 | - | - | - | - | - | - | 0.05 | - | - | - | - | - | - | - |
| CBM50 | - | - | - | - | - | 1.86 | 1.58 | 4.96 | 1.14 | - | 1.37 | 6.41 | - | 1.27 |
| CBM51 | - | - | - | - | - | 0.47 | 0.64 | 1.29 | - | - | - | - | - | 0.73 |
| CBM6 | - | - | - | - | 4.35 | - | 0.30 | 0.65 | 1.14 | - | - | - | - | 2.91 |
| CE11 | 7.27 | 12.92 | - | 2.70 | 13.04 | - | 1.03 | 2.80 | 3.41 | - | 2.74 | 5.13 | - | 0.91 |
| CE14 | - | - | - | 5.41 | 39.13 | - | 0.44 | 14.01 | - | - | 4.11 | - | - | 0.36 |
| CE4 | 1.82 | 5.62 | 2.41 | 10.81 | 21.74 | 12.09 | 6.50 | 17.03 | 9.09 | - | 36.99 | 28.21 | 8.33 | 6.00 |
| CE8 | 1.82 | 0.56 | - | 2.70 | - | - | 2.17 | - | 1.14 | - | - | - | - | - |
| GH1 | 9.09 | 11.52 | 2.41 | 18.92 | - | 26.51 | 14.67 | 4.09 | - | 5.88 | 2.74 | - | 8.33 | 4.91 |
| GH10 | 5.45 | 1.69 | - | - | - | - | 1.13 | 3.88 | 3.41 | 1.18 | - | 1.28 | - | 7.64 |
| GH100 | - | - | - | - | - | - | - | 0.43 | - | - | - | - | - | - |
| GH102 | - | - | 4.82 | 2.70 | 4.35 | - | 0.15 | 0.65 | - | - | 8.22 | 5.13 | 16.67 | - |
| GH11 | - | 0.28 | - | - | - | - | - | - | - | - | - | - | - | 2.73 |
| GH12 | - | - | 1.20 | - | - | - | - | 0.43 | - | - | - | - | - | - |
| GH16 | - | 0.28 | - | - | - | - | 1.23 | 2.16 | 1.14 | - | 1.37 | - | - | 0.91 |
| GH18 | - | 0.84 | - | 2.70 | - | 2.33 | 1.92 | 1.51 | - | - | 2.74 | - | - | 4.91 |
| GH19 | - | - | - | - | - | - | - | 0.65 | - | - | 1.37 | 1.28 | - | 0.18 |
| GH2 | 3.64 | - | - | - | - | 0.47 | 0.39 | 0.22 | - | - | - | - | - | 0.36 |
| GH20 | 25.45 | 9.27 | 2.41 | 2.70 | - | 4.65 | 9.80 | 4.74 | 3.41 | - | 4.11 | 8.97 | 16.67 | 2.91 |
| GH24 | - | - | - | 2.70 | - | - | 0.10 | 0.22 | 2.27 | 1.18 | 4.11 | 8.97 | - | - |
| GH25 | - | 0.28 | - | - | - | 5.12 | 3.69 | 2.37 | - | 3.53 | 2.74 | 2.56 | - | 0.73 |
| GH26 | - | 0.28 | - | 5.41 | 4.35 | 0.47 | 1.38 | 1.08 | 1.14 | 1.18 | - | 1.28 | - | 2.36 |
| GH27 | - | 0.84 | - | - | - | 0.93 | 1.23 | - | - | - | - | - | - | 0.73 |
| GH30 | - | 0.28 | - | 2.70 | - | 0.93 | 1.28 | 1.94 | - | - | 1.37 | 7.69 | - | 0.36 |
| GH31 | 10.91 | 18.26 | 9.64 | 10.81 | - | 14.88 | 13.34 | 5.17 | 7.95 | 2.35 | 9.59 | 10.26 | 25.00 | 6.55 |
| GH35 | 1.82 | 1.97 | 16.87 | - | - | 1.40 | 1.33 | 1.29 | - | - | - | 1.28 | - | 1.09 |
| GH37 | - | - | - | 2.70 | - | - | 0.44 | 0.86 | - | - | 2.74 | - | 8.33 | - |
| GH39 | - | 1.40 | - | - | - | 0.93 | 0.79 | 2.59 | - | - | - | - | - | 2.00 |
| GH4 | 3.64 | 5.62 | 18.07 | 5.41 | - | 6.98 | 3.20 | 4.96 | - | 1.18 | - | - | - | 6.18 |
| GH42 | 1.82 | 4.78 | 10.84 | - | - | 7.44 | 4.68 | 1.72 | - | - | - | 1.28 | - | 5.45 |
| GH45 | - | - | - | - | - | - | - | - | - | - | - | - | - | 0.36 |
| GH47 | - | - | - | - | - | - | - | 0.22 | - | - | - | - | - | - |
| GH48 | - | 0.28 | - | - | - | - | - | - | - | - | - | - | - | - |
| GH5 | - | 0.84 | - | - | - | 1.40 | 2.17 | 2.59 | - | - | 2.74 | - | - | 15.27 |
| GH52 | - | - | - | - | - | - | - | 0.22 | - | - | - | - | - | 0.55 |
| GH57 | - | 1.40 | - | 5.41 | 8.70 | - | 0.79 | 2.80 | 2.27 | - | 2.74 | - | - | 6.55 |
| GH59 | - | - | - | - | - | - | - | 0.22 | - | - | - | - | - | - |

| **CAZy** | **CHC** | **CRU** | **FSH** | **LFG** | **GWO** | **HGG** | **HGK** | **MNS** | **MLE** | **MOB** | **SOZ** | **SUS** | **THG** | **WHF** |
| --- | --- | --- | --- | --- | --- | --- | --- | --- | --- | --- | --- | --- | --- | --- |
| GH6 | - | - | - | - | - | - | - | 1.51 | - | - | - | - | - | - |
| GH61 | - | - | 1.20 | - | - | - | - | - | - | - | - | - | - | - |
| GH62 | - | - | - | - | - | - | - | 0.22 | - | - | - | - | - | - |
| GH63 | - | - | 3.61 | - | - | - | 0.39 | 0.65 | - | - | 1.37 | 2.56 | - | - |
| GH65 | 3.64 | 3.09 | - | 5.41 | - | 0.47 | 2.17 | 1.72 | - | - | - | - | 16.67 | 2.00 |
| GH67 | - | - | - | - | - | - | - | - | - | - | - | - | - | 0.36 |
| GH68 | - | - | 4.82 | - | - | - | 0.05 | - | 52.27 | 80.00 | - | - | - | - |
| GH70 | - | - | - | - | - | - | 0.25 | - | - | - | - | - | - | - |
| GH73 | 3.64 | - | - | - | 4.35 | 0.93 | 1.23 | 0.65 | - | 1.18 | 2.74 | 6.41 | - | 0.18 |
| GH76 | - | - | - | - | - | - | 0.98 | - | - | - | - | - | - | - |
| GH78 | 10.91 | 13.48 | 18.07 | - | - | 6.51 | 8.27 | 2.37 | 10.23 | 1.18 | - | 1.28 | - | 0.91 |
| GH79 | - | - | - | - | - | 0.47 | - | 0.22 | - | - | - | - | - | - |
| GH8 | - | - | 2.41 | 8.11 | - | 0.47 | 0.74 | 0.22 | - | - | - | - | - | 3.27 |
| GH81 | - | - | - | - | - | - | 0.10 | 0.22 | - | - | - | - | - | - |
| GH85 | - | - | - | - | - | - | 0.15 | - | - | - | - | - | - | - |
| GH89 | 9.09 | 3.09 | - | - | - | - | 1.67 | 0.22 | - | - | - | - | - | 0.18 |
| GH9 | - | 1.12 | - | - | - | - | 0.64 | 1.29 | - | - | 1.37 | - | - | 6.18 |
| PL1 | - | - | - | 2.70 | - | - | 0.20 | 1.29 | - | - | - | - | - | 1.64 |
| PL5 | - | - | 1.20 | - | - | - | - | - | - | - | - | - | - | - |
| PL8 | - | - | - | - | - | - | 0.69 | 0.22 | - | 1.18 | - | - | - | - |
