# Supplementary material for: An Insect Herbivore Microbiome with High Plant Biomass-Degrading Capacity
Source: PLoS Genet. 2010 Sep 23;6(9):e1001129. doi: 10.1371/journal.pgen.1001129 (PMC2944797; doi:10.1371/journal.pgen.1001129)
Supplement: Table S10 — Gene category distribution of the bacterial portion of the leaf-cutter ant fungus garden metagenome as annotated using clusters of orthologous groups (COGs). A total of 8,092 ORFs (or ∼50%) out of 16,342 predicted bacterial ORFs in the fungus garden community metagenome was annotated to a COG category, as shown. The % of annotated ORFs for each COG category is also shown. (0.06 MB DOC) [file pgen.1001129.s024.doc]

| **COG Category** | **Code** | **No. COGs** | **% Annotated COGs** |
| --- | --- | --- | --- |
| **Information storage and processing** | | | |
| Translation, ribosomal structure and biogenesis | J | 385 | 4.76% |
| RNA processing and modification | A | 3 | 0.04% |
| Transcription | K | 495 | 6.12% |
| Replication, recombination and repair | L | 415 | 5.13% |
| Chromatin structure and dynamics | B | 3 | 0.04% |
| **Cellular processes and signaling** | | | |
| Cell cycle control, cell division, chromosome partitioning | D | 73 | 0.90% |
| Nuclear structure | Y | 0 | 0.00% |
| Defense mechanisms | V | 220 | 2.72% |
| Signal transduction mechanisms | T | 490 | 6.06% |
| Cell wall/membrane/envelope biogenesis | M | 447 | 5.52% |
| Cell motility | N | 123 | 1.52% |
| Cytoskeleton | Z | 3 | 0.04% |
| Extracellular structures | W | 0 | 0.00% |
| Intracellular trafficking, secretion, and vesicular transport | U | 231 | 2.85% |
| Posttranslational modification, protein turnover, chaperones | O | 301 | 3.72% |
| **Metabolism** | | | |
| Energy production and conversion | C | 731 | 9.03% |
| Carbohydrate transport and metabolism | G | 653 | 8.07% |
| Amino acid transport and metabolism | E | 1066 | 13.17% |
| Nucleotide transport and metabolism | F | 221 | 2.73% |
| Coenzyme transport and metabolism | H | 310 | 3.83% |
| Lipid transport and metabolism | I | 298 | 3.68% |
| Inorganic ion transport and metabolism | P | 523 | 6.46% |
| Secondary metabolites biosynthesis, transport and catabolism | Q | 238 | 2.94% |
| **Poorly characterized** | | | |
| General function prediction only | R | 924 | 11.42% |
| Function unknown | S | 576 | 7.12% |
