# Supplementary material for: An Insect Herbivore Microbiome with High Plant Biomass-Degrading Capacity
Source: PLoS Genet. 2010 Sep 23;6(9):e1001129. doi: 10.1371/journal.pgen.1001129 (PMC2944797; doi:10.1371/journal.pgen.1001129)
Supplement: Table S11 — Identified COGs in the leaf-cutter ant fungus garden metagenome that belong to secondary metabolites biosynthesis, transport and catabolism (Q) category. The COG ID, total identified number, and COG annotation are shown. (0.08 MB DOC) [file pgen.1001129.s025.doc]

| **COGID** | **Number** | **Annotation** |
| --- | --- | --- |
| COG0123 | 3 | Deacetylases, including yeast histone deacetylase and acetoin utilization protein |
| COG0145 | 12 | N-methylhydantoinase A/acetone carboxylase, beta subunit |
| COG0146 | 8 | N-methylhydantoinase B/acetone carboxylase, alpha subunit |
| COG0179 | 7 | 2-keto-4-pentenoate hydratase/2-oxohepta-3-ene-1,7-dioic acid hydratase (catechol pathway) |
| COG0304 | 10 | 3-oxoacyl-(acyl-carrier-protein) synthase |
| COG0318 | 42 | Acyl-CoA synthetases (AMP-forming)/AMP-acid ligases II |
| COG0412 | 9 | Dienelactone hydrolase and related enzymes |
| COG0767 | 5 | ABC-type transport system involved in resistance to organic solvents, permease component |
| COG1020 | 17 | Non-ribosomal peptide synthetase modules and related proteins |
| COG1021 | 1 | Peptide arylation enzymes |
| COG1127 | 5 | ABC-type transport system involved in resistance to organic solvents, ATPase component |
| COG1228 | 9 | Imidazolonepropionase and related amidohydrolases |
| COG1233 | 2 | Phytoene dehydrogenase and related proteins |
| COG1335 | 8 | Amidases related to nicotinamidase |
| COG1463 | 2 | ABC-type transport system involved in resistance to organic solvents, periplasmic component |
| COG1535 | 2 | Isochorismate hydrolase |
| COG2015 | 1 | Alkyl sulfatase and related hydrolases |
| COG2050 | 3 | Uncharacterized protein, possibly involved in aromatic compounds catabolism |
| COG2124 | 12 | Cytochrome P450 |
| COG2132 | 6 | Putative multicopper oxidases |
| COG2162 | 1 | Arylamine N-acetyltransferase |
| COG2175 | 7 | Probable taurine catabolism dioxygenase |
| COG2313 | 1 | Uncharacterized enzyme involved in pigment biosynthesis |
| COG2368 | 2 | Aromatic ring hydroxylase |
| COG2508 | 1 | Regulator of polyketide synthase expression |
| COG2761 | 1 | Predicted dithiol-disulfide isomerase involved in polyketide biosynthesis |
| COG2854 | 3 | ABC-type transport system involved in resistance to organic solvents, auxiliary component |
| COG3127 | 4 | Predicted ABC-type transport system involved in lysophospholipase L1 biosynthesis, permease component |
| COG3155 | 1 | Uncharacterized protein involved in an early stage of isoprenoid biosynthesis |
| COG3191 | 1 | L-aminopeptidase/D-esterase |
| COG3208 | 1 | Predicted thioesterase involved in non-ribosomal peptide biosynthesis |
| COG3284 | 1 | Transcriptional activator of acetoin/glycerol metabolism |
| COG3315 | 2 | O-Methyltransferase involved in polyketide biosynthesis |
| COG3320 | 1 | Putative dehydrogenase domain of multifunctional non-ribosomal peptide synthetases and related enzymes |
| COG3321 | 5 | Polyketide synthase modules and related proteins |
| COG3435 | 5 | Gentisate 1,2-dioxygenase |
| COG3485 | 3 | Protocatechuate 3,4-dioxygenase beta subunit |
| COG3508 | 1 | Homogentisate 1,2-dioxygenase |
| COG3509 | 2 | Poly(3-hydroxybutyrate) depolymerase |
| COG3648 | 1 | Uricase (urate oxidase) |
| COG3653 | 8 | N-acyl-D-aspartate/D-glutamate deacylase |
| COG3670 | 1 | Lignostilbene-alpha,beta-dioxygenase and related enzymes |
| COG3733 | 2 | Cu2+-containing amine oxidase |
| COG3917 | 2 | 2-hydroxychromene-2-carboxylate isomerase |
| COG3971 | 3 | 2-keto-4-pentenoate hydratase |
| COG4576 | 1 | Carbon dioxide concentrating mechanism/carboxysome shell protein |
| COG4577 | 1 | Carbon dioxide concentrating mechanism/carboxysome shell protein |
| COG4663 | 4 | TRAP-type mannitol/chloroaromatic compound transport system, periplasmic component |
| COG4664 | 3 | TRAP-type mannitol/chloroaromatic compound transport system, large permease component |
| COG4665 | 2 | TRAP-type mannitol/chloroaromatic compound transport system, small permease component |
| COG4689 | 1 | Acetoacetate decarboxylase |
| COG4909 | 1 | Propanediol dehydratase, large subunit |
| COG5517 | 1 | Small subunit of phenylpropionate dioxygenase |
