# Supplementary material for: An Insect Herbivore Microbiome with High Plant Biomass-Degrading Capacity
Source: PLoS Genet. 2010 Sep 23;6(9):e1001129. doi: 10.1371/journal.pgen.1001129 (PMC2944797; doi:10.1371/journal.pgen.1001129)
Supplement: Table S12 — Draft genome characteristics of the leaf-cutter ant-associated nitrogen-fixing bacterial symbionts Pantoea sp. At-9b and Klebsiella variicola At-22. (0.03 MB DOC) [file pgen.1001129.s026.doc]

| **Characteristic** | ***Pantoea* sp. At-9b** | ***Klebsiella variicola At-22*** |
| --- | --- | --- |
| Draft genome size | 6,254,009 bp | 5,407,846 bp |
| Contigs | 90 | 36 |
| Largest contig | 423,458 bp | 796,270 bp |
| Average contig size | 69,489 bp | 150,217 |
| G+C% | 54.30% | 57.60% |
| Predicted ORFs | 5,959 | 5,055 |
