# Supplementary material for: An Insect Herbivore Microbiome with High Plant Biomass-Degrading Capacity
Source: PLoS Genet. 2010 Sep 23;6(9):e1001129. doi: 10.1371/journal.pgen.1001129 (PMC2944797; doi:10.1371/journal.pgen.1001129)
Supplement: Table S13 — Carbohydrate-active enzyme (CAZy) annotation of the predicted proteome of Klebsiella variicola At-22. Only those proteins that had a significant hit (e-value < 1e-05) to an enzyme in the CAZy database and to each CAZy family's associated protein domain (Pfam) annotation were retained. Specifically, the locus, predicted CAZy family, and top BLAST hit (including closest matching organism) are provided below. (0.07 MB DOC) [file pgen.1001129.s027.doc]

| **Locus** | **CAZy Family** | **Top BLAST Hit** |
| --- | --- | --- |
| KvarDRAFT_2014 | CBM34 | maltodextrin glucosidase [Klebsiella pneumoniae 342] |
| KvarDRAFT_4985 | CBM50 | membrane-bound lytic murein transglycosylase D [Klebsiella pneumoniae 342] |
| KvarDRAFT_2883 | CE11 | UDP-3-O-acyl N-acetylglucosamine deacetylase  [Klebsiella pneumoniae subsp. pneumoniae MGH 78578] |
| KvarDRAFT_1588 | CE14 | LmbE family protein [Natranaerobius thermophilus JW/NM-WN-LF] |
| KvarDRAFT_0589 | CE4 | putative urate catabolism protein [Klebsiella pneumoniae 342] |
| KvarDRAFT_2921 | CE4 | polysaccharide deacetylase domain protein [Klebsiella pneumoniae 342] |
| KvarDRAFT_3746 | CE8 | pectinesterase [Klebsiella pneumoniae 342] |
| KvarDRAFT_0792 | GH1 | 6-phospho-beta-glucosidase BglA [Klebsiella pneumoniae 342] |
| KvarDRAFT_0762 | GH1 | 6-phospho-beta-glucosidase pbgA [Klebsiella pneumoniae 342] |
| KvarDRAFT_0576 | GH1 | beta-glucosidase [Klebsiella pneumoniae 342] |
| KvarDRAFT_3933 | GH1 | 6-phospho-beta-glucosidase BglB [Klebsiella pneumoniae 342] |
| KvarDRAFT_0947 | GH102 | membrane-bound lytic murein transglycosylase A [Klebsiella pneumoniae 342] |
| KvarDRAFT_4283 | GH19 | putative glycoside hydrolase [Klebsiella pneumoniae MGH 78578] |
| KvarDRAFT_0375 | GH2 | beta-galactosidase [Klebsiella pneumoniae 342] |
| KvarDRAFT_1400 | GH24 | phage lysozyme [Klebsiella pneumoniae 342] |
| KvarDRAFT_1619 | GH31 | alpha-xylosidase [Klebsiella pneumoniae 342] |
| KvarDRAFT_1768 | GH37 | putative trehalase [Klebsiella pneumoniae 342] |
| KvarDRAFT_1494 | GH37 | trehalase [Klebsiella pneumoniae 342] |
| KvarDRAFT_0029 | GH4 | 6-phospho-beta-glucosidase [Klebsiella pneumoniae 342] |
| KvarDRAFT_4573 | GH4 | maltose-6'-phosphate glucosidase [Klebsiella pneumoniae 342] |
| KvarDRAFT_0466 | GH4 | maltose-6'-phosphate glucosidase [Klebsiella pneumoniae 342] |
| KvarDRAFT_3110 | GH4 | alpha-galactosidase [Klebsiella pneumoniae 342] |
| KvarDRAFT_2100 | GH42 | beta-galactosidase [Klebsiella pneumoniae 342] |
| KvarDRAFT_0684 | GH78 | alpha-L-rhamnosidase family protein [Klebsiella pneumoniae 342] |
| KvarDRAFT_1756 | GH8 | cellulase [Klebsiella pneumoniae 342] |
| KvarDRAFT_2978 | GT19 | lipid-A-disaccharide synthase [Klebsiella pneumoniae 342] |
| KvarDRAFT_2877 | GT28 | undecaprenyldiphospho-muramoylpentapeptide beta-N-acetylglucosaminyltransferase [Klebsiella pneumoniae 342] |
| KvarDRAFT_1850 | GT35 | maltodextrin phosphorylase [Klebsiella pneumoniae 342] |
| KvarDRAFT_1842 | GT35 | glycogen phosphorylase [Klebsiella pneumoniae 342] |
| KvarDRAFT_3883 | GT35 | glycogen/starch/alpha-glucan phosphorylase family protein  [Klebsiella pneumoniae 342] |
| KvarDRAFT_1870 | GT51 | penicillin-binding protein 1A [Klebsiella pneumoniae 342] |
| KvarDRAFT_2947 | GT51 | penicillin-binding protein 1B [Klebsiella pneumoniae 342] |
| KvarDRAFT_2312 | GT51 | penicillin-binding protein 1C [Klebsiella pneumoniae 342] |
| KvarDRAFT_2578 | GT51 | monofunctional biosynthetic peptidoglycan transglycosylase  [Klebsiella pneumoniae 342] |
| KvarDRAFT_1675 | GT9 | lipopolysaccharide heptosyltransferase II [Klebsiella pneumoniae 342] |
| KvarDRAFT_1674 | GT9 | lipopolysaccharide heptosyltransferase I [Klebsiella pneumoniae 342] |
| KvarDRAFT_1669 | GT9 | lipopolysaccharide core biosynthesis glycosyltransferase RfaQ  [Klebsiella pneumoniae 342] |
| KvarDRAFT_4113 | GT9 | heptosyltransferase family protein [Klebsiella pneumoniae 342] |
