# Supplementary material for: An Insect Herbivore Microbiome with High Plant Biomass-Degrading Capacity
Source: PLoS Genet. 2010 Sep 23;6(9):e1001129. doi: 10.1371/journal.pgen.1001129 (PMC2944797; doi:10.1371/journal.pgen.1001129)
Supplement: Table S14 — Carbohydrate-active enzyme (CAZy) annotation of the predicted proteome of Pantoea sp. At-9b. Only those proteins that had a significant hit (e-value < 1e-05) to an enzyme in the CAZy database and to each CAZy family's associated protein domain (Pfam) annotation were retained. Specifically, the locus, predicted CAZy family, and top BLAST hit (including closest matching organism) are provided below. (0.06 MB DOC) [file pgen.1001129.s028.doc]

| **Locus** | **CAZy Family** | **Top BLAST Hit** |
| --- | --- | --- |
| Pat9bDRAFT_3621 | CBM33 | chitin-binding protein [Myxococcus xanthus DK 1622] |
| Pat9bDRAFT_5520 | CBM50 | Membrane-bound lytic murein transglycosylase D  [Erwinia tasmaniensis Et1/99] |
| Pat9bDRAFT_4117 | CBM50 | N-acetylmuramoyl-L-alanine amidase [Aliivibrio salmonicida LFI1238] |
| Pat9bDRAFT_3107 | CE11 | UDP-3-O-[3-hydroxymyristoyl] N-acetylglucosamine deacetylase  [Enterobacter sp. 638] |
| Pat9bDRAFT_0093 | CE14 | LmbE family protein [Natranaerobius thermophilus JW/NM-WN-LF] |
| Pat9bDRAFT_5672 | CE4 | polysaccharide deacetylase [Azorhizobium caulinodans ORS 571] |
| Pat9bDRAFT_5529 | CE4 | Putative polysaccharide deacetylase [Erwinia tasmaniensis Et1/99] |
| Pat9bDRAFT_3240 | CE4 | polysaccharide deacetylase family protein  [Pseudomonas syringae pv. phaseolicola 1448A] |
| Pat9bDRAFT_2095 | CE4 | Polysaccharide deacetylase [Pseudomonas fluorescens Pf0-1] |
| Pat9bDRAFT_2096 | CE4 | Polysaccharide deacetylase [Pseudomonas fluorescens Pf0-1] |
| Pat9bDRAFT_0205 | GH1 | glycoside hydrolase family 1 [Serratia proteamaculans 568] |
| Pat9bDRAFT_4825 | GH1 | glycoside hydrolase family 1 [Serratia proteamaculans 568] |
| Pat9bDRAFT_3476 | GH1 | beta-glucosidase [Klebsiella pneumoniae 342] |
| Pat9bDRAFT_4819 | GH1 | putative glycosyl phosphatase [Escherichia fergusonii] |
| Pat9bDRAFT_4129 | GH1 | 6-phospho-beta-glucosidase [Pectobacterium atrosepticum SCRI1043] |
| Pat9bDRAFT_5495 | GH102 | Membrane-bound lytic murein transglycosylase A  [Erwinia tasmaniensis Et1/99] |
| Pat9bDRAFT_1746 | GH18 | chitinase [Bombyx mori] |
| Pat9bDRAFT_2680 | GH19 | putative glycoside hydrolase [Klebsiella pneumoniae MGH 78578] |
| Pat9bDRAFT_0726 | GH2 | Beta-galactosidase (Lactase) [Erwinia tasmaniensis Et1/99] |
| Pat9bDRAFT_3337 | GH20 | beta-N-acetylhexosaminidase [Enterobacter sp. 638] |
| Pat9bDRAFT_3807 | GH24 | Putative phage lysozyme [Erwinia tasmaniensis Et1/99] |
| Pat9bDRAFT_1775 | GH37 | Periplasmic trehalase [Erwinia tasmaniensis Et1/99] |
| Pat9bDRAFT_5690 | GH8 | endoglucanase [Erwinia rhapontici] |
| Pat9bDRAFT_4892 | GT19 | lipid-A-disaccharide synthase [Enterobacter sp. 638] |
| Pat9bDRAFT_3987 | GT26 | probable UDP-N-acetyl-D-mannosaminuronic acid transferase  [Pectobacterium atrosepticum SCRI1043] |
| Pat9bDRAFT_5095 | GT35 | Glycogen phosphorylase [Erwinia tasmaniensis Et1/99] |
| Pat9bDRAFT_4434 | GT35 | maltodextrin phosphorylase [Klebsiella pneumoniae 342] |
| Pat9bDRAFT_4177 | GT51 | Monofunctional biosynthetic peptidoglycan transglycosylase  [Erwinia tasmaniensis Et1/99] |
| Pat9bDRAFT_4860 | GT51 | Penicillin-binding protein 1B [Erwinia tasmaniensis Et1/99] |
| Pat9bDRAFT_4416 | GT51 | Penicillin-binding protein 1A [Erwinia tasmaniensis Et1/99] |
| Pat9bDRAFT_3684 | GT9 | putative glycosyl transferase [Serratia proteamaculans 568] |
| Pat9bDRAFT_3691 | GT9 | glycosyl transferase family 9 [Serratia proteamaculans 568] |
| Pat9bDRAFT_3685 | GT9 | Heptosyltransferase I WaaC [Erwinia tasmaniensis Et1/99] |
| Pat9bDRAFT_3686 | GT9 | ADP-heptose: LPS heptosyltransferase II [Escherichia coli O127:H6] |
