# Supplementary material for: An Insect Herbivore Microbiome with High Plant Biomass-Degrading Capacity
Source: PLoS Genet. 2010 Sep 23;6(9):e1001129. doi: 10.1371/journal.pgen.1001129 (PMC2944797; doi:10.1371/journal.pgen.1001129)
Supplement: Table S15 — Cellulose-degradation bioassays for Klebsiella variicola At-22 and Pantoea sp. At-9b. Cultures of both bacteria were grown on carboxymethyl cellulose or microcrystalline. Confirmation of this assay was done by growing these cultures using only crystalline cellulose (CMC) or microcrystalline cellulose as a carbon source. CMC data is reported as the area zone of clearing when assayed using Congo Red (mm2). Microcrystalline cellulose growth is reported as either a plus (+) or minus (−) indicating positive or negative results for growth. (0.03 MB DOC) [file pgen.1001129.s029.doc]

| **Bacterial Isolate** | **Growth on Carboxymethyl**  **Cellulose (mm^2^)** | **Growth on Microcrystalline Cellulose** |
| --- | --- | --- |
| *Klebsiella* *variicola* At-22 | 19.7 ± 5.21 | – |
| *Pantoea* sp. At-9b | 12.2 ± 0.115 | + |
