# Supplementary material for: An Insect Herbivore Microbiome with High Plant Biomass-Degrading Capacity
Source: PLoS Genet. 2010 Sep 23;6(9):e1001129. doi: 10.1371/journal.pgen.1001129 (PMC2944797; doi:10.1371/journal.pgen.1001129)
Supplement: Table S16 — Recruitment analysis of the leaf-cutter ant fungus garden community metagenome. Reads from the fungus garden community metagenome were recruited onto complete genomes in the prokaryotic genome collection in addition to the draft genomes of Klebsiella variicola At-22 and Pantoea sp. At-9b generated in this study. Only those organisms with more than 100 recruited reads are shown. The total number of recruited reads, the number of reads with >98% sequence identity, and the corresponding percentage is shown. (0.05 MB DOC) [file pgen.1001129.s030.doc]

| **Organism** | **Total Mapped Reads** | **Total Reads > 98% Sequence Identity** |
| --- | --- | --- |
| *Pantoea sp. At-9b* | 2,064 | 81 (3.92%) |
| *Escherichia coli SE11* | 991 | 877 (88.50%) |
| *Klebsiella variicola At-22* | 590 | 528 (89.49%) |
| *Klebsiella pneumoniae MGH 78578* | 347 | 172 (49.57%) |
| *Serratia proteamaculans 568* | 306 | 1 (0.33%) |
| *Enterobacter 638* | 293 | 4 (1.37%) |
| *Klebsiella pneumoniae 342* | 289 | 195 (67.47%) |
| *Sulfolobus acidocaldarius DSM 639* | 271 | 0 (0.00%) |
| *Escherichia coli 55989* | 266 | 244 (91.73%) |
| *Nitrosopumilus maritimus SCM1* | 262 | 2 (0.76%) |
| *Bacillus cereus ZK* | 238 | 0 (0.00%) |
| *Ignicoccus hospitalis KIN4 I* | 233 | 0 (0.00%) |
| *Trichodesmium erythraeum IMS101* | 233 | 0 (0.00%) |
| *Erwinia tasmaniensis* | 229 | 1 (0.44%) |
| *Candidatus Sulcia muelleri GWSS* | 204 | 1 (0.49%) |
| *Thermococcus onnurineus NA1* | 194 | 9 (4.64%) |
| *Cyanothece ATCC 51142* | 188 | 0 (0.00%) |
| *Bradyrhizobium japonicum* | 183 | 2 (1.09%) |
| *Enterobacter sakazakii ATCC BAA-894* | 170 | 3 (1.76%) |
| *Salmonella typhi* | 163 | 129 (79.14%) |
| *Burkholderia xenovorans LB400* | 149 | 60 (40.27%) |
| *Solibacter usitatus Ellin6076* | 140 | 2 (1.43%) |
| *Microcystis aeruginosa NIES 843* | 140 | 0 (0.00%) |
| *Cyanothece PCC 7424* | 139 | 0 (0.00%) |
| *Thermoplasma volcanium* | 129 | 0 (0.00%) |
| *Methanococcus aeolicus Nankai-3* | 124 | 0 (0.00%) |
| *Saccharopolyspora erythraea NRRL 2338* | 108 | 2 (0.00%) |
| *Methanosphaera stadtmanae* | 107 | 101 (94.39%) |
| *Escherichia coli ED1a* | 107 | 0 (0.00%) |
| *Streptomyces griseus NBRC 13350* | 106 | 53 (50.00%) |
| *Burkholderia phymatum STM815* | 100 | 69 (69.00%) |
