# Supplementary material for: An Insect Herbivore Microbiome with High Plant Biomass-Degrading Capacity
Source: PLoS Genet. 2010 Sep 23;6(9):e1001129. doi: 10.1371/journal.pgen.1001129 (PMC2944797; doi:10.1371/journal.pgen.1001129)
Supplement: Text S1 — GC Content Analysis of the Community Metagenome. (0.03 MB DOC) [file pgen.1001129.s031.doc]

Text S1. GC Content Analysis of the Community Metagenome.

GC content analysis of the bacterial portion of the leaf-cutter ant fungus garden community metagenome confirmed that this microbiome is dominated by bacteria in the γ-proteobacteria, α-proteobacteria, Actinobacteria, and β-proteobacteria (Figure S11). The Actinobacteria had the highest average GC content, characteristic of the GC content observed for sequenced genomes in this phylum. GC content analysis of the eukaryotic portion of the community metagenome was performed specifically for those sequences identified as belonging to fungi, plants, or metazoan (Figure S12). Interestingly, GC content analysis of the fungi revealed two distinct GC peaks, which we speculate may correspond to different populations of fungi. It is unclear what the first peak at ~34% represents, as no sequenced fungal genome that is closely-related to a known fungal symbiont of these ants (e.g. *Leucoagaricus*, *Escovopsis*) has a comparable average GC. The second peak likely corresponds to the fungus the ants cultivate for food, as the 45% GC is similar to the GC of a closely-related fungus for which a genome sequence exists, *Laccaria bicolor*, which has a GC content of ~47% [1]. Analysis of the unclassified portion of the community metagenome revealed that it has a large single peak at ~30% (Figure S12). This may indicate sequences corresponding to the unknown fungal group, plants the ants harvest, or perhaps even the ant itself.

**References**

1. Martin F, Aerts A, Ahren D, Brun A, Danchin EG, et al. (2008) The genome of Laccaria bicolor provides insights into mycorrhizal symbiosis. Nature 452: 88-92.
