# Supplementary material for: An Insect Herbivore Microbiome with High Plant Biomass-Degrading Capacity
Source: PLoS Genet. 2010 Sep 23;6(9):e1001129. doi: 10.1371/journal.pgen.1001129 (PMC2944797; doi:10.1371/journal.pgen.1001129)
Supplement: Text S2 — COG Clustering Analysis of the Community Metagenome. (0.04 MB DOC) [file pgen.1001129.s032.doc]

Text S2. COG Clustering Analysis of the Community Metagenome.

We analyzed the gene content of the fungus garden community metagenome using clusters of orthologous groups (COGs [1]) (Table S10) and compared this profile, using clustering analysis [2], to the COG profiles of 13 other published metagenomes. This analysis revealed that the fungus garden metagenome groups closest to the metagenomes of Minnesota soil [2], whale fall [2], and wastewater sludge [3] (Figure 3B). Gene category assessment of these metagenomes shows they share similar gene proportions, with the highest categories corresponding to amino acid transport and metabolism (10%); energy production and conversion (8%); and inorganic ion transport and metabolism (6%) (Figure S13). Interestingly, the leaf-cutter ant fungus garden metagenome had the highest proportion of genes associated with secondary metabolite biosynthesis, transport and catabolism when compared to all other metagenomes. Analysis of these genes indicate that many are associated with polyketide and non-ribosomal peptide production (Table S11), perhaps reflecting an important role for these molecules in shaping symbiont interactions within this community [4,5].

**References**

1. Tatusov RL, Galperin MY, Natale DA, Koonin EV (2000) The COG database: a tool for genome-scale analysis of protein functions and evolution. Nucleic Acids Res 28: 33-36.

**2. Tringe SG, von Mering C, Kobayashi A, Salamov AA, Chen K, et al. (2005) Comparative metagenomics of microbial communities. Science 308: 554-557.**

**3. Garcia Martin H, Ivanova N, Kunin V, Warnecke F, Barry KW, et al. (2006) Metagenomic analysis of two enhanced biological phosphorus removal (EBPR) sludge communities. Nat Biotechnol 24: 1263-1269.**

**4. Haeder S, Wirth R, Herz H, Spiteller D (2009) Candicidin-producing Streptomyces support leaf-cutting ants to protect their fungus garden against the pathogenic fungus Escovopsis. Proc Natl Acad Sci U S A 106: 4742-4746.**

**5. Oh DC, Poulsen M, Currie CR, Clardy J (2009) Dentigerumycin: a bacterial mediator of an ant-fungus symbiosis. Nat Chem Biol 5: 391-393.**
